# An analysis of crude oil prices in the last decade (2011-2020): With deep learning approach

**DOI:** 10.1371/journal.pone.0268996

**Published:** 2023-03-09

**Authors:** Abhibasu Sen, Karabi Dutta Choudhury, Tapan Kumar Datta

**Affiliations:** 1 Department of Mathematics, Assam University, Dargakona, Assam, India; 2 Department of Mathematics, Institute of Technology,Nirma University, Gujarat, India; Institute for Economic Forecasting, Romanian Academy, ROMANIA

## Abstract

Crude Oil is one of the most important commodities in this world. We have studied the effects of Crude Oil inventories on crude oil prices over the last ten years (2011 to 2020). We tried to figure out how the Crude Oil price variance responds to inventory announcements. We then introduced several other financial instruments to study the relation of these instruments with Crude Oil variation. To undertake this task, we took the help of several mathematical tools including machine learning tools such as Long Short Term Memory(LSTM) methods, etc. The previous researches in this area primarily focussed on statistical methods such as GARCH (1,1) etc. (Bu (2014)). Various researches on the price of crude oil have been undertaken with the help of LSTM. But the variation of crude oil price has not yet been studied. In this research, we studied the variance of crude oil prices with the help of LSTM. This research will be beneficial for the *options* traders who would like to get benefit from the variance of the underlying instrument.

## 1 Introduction

The price of a commodity generally depends on the demand and supply of the underlying commodity. That is why the amount of inventory of the commodity is an important aspect. Lots of research work have been done to predict commodity prices. Zhang and Na [1] formulated a hybrid *MEA-SVM* method which has higher predictive power for agro-based commodities. Xiong et al. [2] devised a novel hybrid forecasting method on the backdrop of seasonality in vegetable prices for the Chinese markets. Ouyang et al. [3] devised a *Long-Short Time Series Network* to predict global agricultural commodity *futures* price, which is found to be better performing than other methods. Mustaffa et al. [4] used the *Swarm Intelligence* approach to optimize parameters of least squares support vector machines. They found that their strategy performed better than the *Back Propagation Neural Network* and *Genetic Algorithm*. Kohzadi et al. [5] made a comparison of time series models with *artificial neural network (ANN)*, which could make better forecast of the commodity price. The data was monthly live cattle and wheat prices from 1950 to 1990. They found that the neural network model is a better performing one in the above context. Gunawan et al. [6] devised a neural network predicting method for Indonesian Palm Oil. They concluded that by increasing the number of iterations, it could increase accuracy but it comes at the cost of computational overheads. Gargano and Timmermann [7] forecasted the commodity price indices using macroeconomic and financial indicators. They found that commodity prices could better predict at monthly or quarterly time scale while macroeconomic indicators could better predict at a yearly time scale. They found that commodity prices could be best predicted during economic recessions. Claussen and Uhrig [8] found that neural networks could better forecast the directional changes of the price of cash soybean oil compared to other statistical tests. Chen and Wang [9] forecasted natural rubber prices using *Genetic Fuzzy logic* and *Fuzzy logic*. They found out that Genetic Fuzzy logic gives better results than Fuzzy logic. Cash Soybean Oil is an agricultural product being actively traded in Chicago Mercantile Exchange (CME). Mahajan [10] found that the *Hybrid Quantum Neural Network* method is better than the *Classical Neural Network* method. Chen et al. [11] devised an application for an automated commodity price prediction system in Malaysia. They compared *ARIMA, SVR, Prophet, XGBoost, and LSTM* and finally found that the LSTM method is the most reliable one. Kulkarni and Haidar [12] formulated an ANN model to forecast spot crude oil prices using *futures* crude oil prices successfully. Parida et al. [13] devised models for forecasting prices of Chana and Barley. The main aim of their research was to determine optimized weights in neural networks using a *Kernel-based Extreme Learning Machine (KELM)*. They compared between *Genetic Algorithm (GA) based KELM (GA-KELM), Particle Swarm Optimization (PSO) based KELM (PSO-KELM), and Grey Wolf Optimization-based multi quadratic kernel KELM (GWO-KELM)*. They found that GWO-KELM produces the best result. Yu et al. [14] forecasted the price of crude oil using a novel decomposition ensemble method. To begin with, they divided the whole data by the *Extended Extreme Learning Machine (EELM)* method. They then predicted the data using machine learning tools. In the end, the predicted results were ensembled to produce the final results.

## 2 Literature review

Hacer et al. [15] tried to forecast the monthly crude oil price using the *bagging ensemble models* and found that classification and regression trees perform better than artificial neural network-based models. With the idea of bagging being introduced, they claimed to have gained better accuracy in both the models. Abdollahi [16] devised a hybrid model for oil price forecasting and it is found to be better performing than other conventional models. Yu et al. [17] made a comparative analysis of five different prediction models and found that *Support Vector Machine (SVM)* is most suitable for crude oil price prediction. In the above work, the SVM model was compared with five other models namely, *feed-forward neural networks (FNN), auto-regressive integrated moving average (ARIMA) model, fractional integrated ARIMA (ARFIMA) model, Markov-switching ARFIMA (MS-ARFIMA) model, and random walk (RW) model*. The directional prediction is highest when the SVM method was used and found to be 74.42%. Sun et al. [18] predicted the interval-valued price of crude oil using the *Interval Decomposition Ensemble method* and found it to be performing better than other predictions. Gao and Lei [19] devised a novel approach to forecast the price of crude oil with a new ‘Machine Learning’ method known as *Stream Learning*. He et al. [20] devised a wavelet-based *Decomposed Ensemble model*, which takes care of the non-stationarity and dynamic changing nature of crude oil prices. Huang and Wang [21] devised a method which combined the *Wavelet Neural Network (WNN)* with random effective function to capture the nonlinearity in the data. They compared it with *Back Propagation Neural Network, SVM, and WNN* and finally found the hybrid model to be more effective than others. Shin et al. [22] predicted the monthly price of crude oil from January 1992 to June 2008, using the *Semi-Supervised Learning (SSL)* method. Chen et al. [23] made a hybrid model based on the deep learning method to forecast the price of crude oil, which they claimed performs better than others. Yu et al. [24] proposed a novel method to forecast the price of crude oil. They at first used *Compressed Sensing based Denoising (CSD)* to filter out the noises therein the data. Then they used the *Artificial Neural Network (ANN) and Least Square Support Vector Regression (LSSVR)* methods to forecast the price of crude oil which is better-performing than other benchmark models. Cao et al. [25] proposed two hybrid models for stock market prices forecasting. They used two kinds of *Empirical Mode Decomposition (EMD)* with *LSTM* for ensembling. They found greater forecasting power with the ensembled method when compared with other methods. Bristone et al. [26] proposed a hybrid model using *Complex Network Analysis (CNN)* and *LSTM*. Gupta and Pandey [27] studied the movements of Crude Oil, Gold and Indian stock index (NIFTY) based on LSTM technique. The prices of Crude Oil and Gold have been taken from Commodity Exchange of India. They could make several successsful predictions based on their work.

Many other research work have also been carried out on forecasting the prices of oil from inventory levels. Hui [28] found out that, *inventory information shocks* negatively affect the *crude oil returns* on the days of inventory announcements by EIA. However, *inventory shocks* do not influence the daily conditional variance. They have also found out that the results of *inventory shocks* do not hold good during rapid growth or decline stage. Miao et al. [29] tried to test the significance of six factors, namely; supply, demand, financial market, commodities market, speculative, and geopolitical; for various forecasting models. These models are used to forecast the price of crude oil. They found that *Least Absolute Shrinkage and Selection Operator (LASSO)* provides the best possible forecast as compared to other methods. Mikhaylov and Moiseev [30] proposed a Machine Learning approach to predict the prices of crude oil by analysing various factors such as US key rate, US dollar index, S&P 500 index, variance index, and US consumer price index. They predicted that the prices of crude oil would have a slight uptrend during 2019-2022. Later on, we can see that the price of crude oil; at the time of writing this article, maintained a steady price level. Ye et al. [31] studied the changing relationships between the prices of crude oil and several other factors. Their study covered the period from January 1992 to December 2007. They found out that prior to 2004, crude oil prices mainly depended on the inventory levels of OECD and excess production capacity of OPEC. They introduced a ratchet variable to indicate the changes in the relationships. The model they formulated had worked well during post-Gulf War I time. For future studies, in the last paragraph they mentioned:

*“Several areas emerge for future studies. First, the changing role of short-term interest rates should be explored in future efforts to improve the model. Second, recent literature,14 as well as market practitioners, also speculates about impacts of exchange rates on crude oil prices, particularly since crude oil is traded in U.S. dollars, which has depreciated significantly against other major currencies since late 2007. Third, OECD inventories had been assumed to be representative of world crude oil inventories in the previous modeling efforts for crude oil markets in the 1990’s. This assumption may no longer be valid, given the rapidly growing economic activity and associated increase in petroleum consumption in the Asia-Pacific region. Finally, the interaction role of the financial market and commodity traders with physical markets needs to be studied to determine any effects on petroleum prices*.*”*

Many other research work have been carried out based on the US-bond yield too. Narayan et al. [32] conducted research comparing the lagged interactions between several economic segments like bonds, equities, commodities, inflations, etc. They found that bond prices negatively *granger cause* crude oil prices. Saenong et al. [33] studied the effect of crude oil prices and exchange rates on the 10-Year bond yields of Indonesia. They found out that in the long run both the crude oil prices and the exchange rate do not affect bond yields, but in the short term they do. Bhar and Lee [34] found out that risk premiums in equity and bond markets can explain the risk premiums in crude oil prices. Coleman [35] investigated the drivers of crude oil price and found out that OPEC market share, corporate bond yields (which are Aaa credited having the highest degree of creditworthiness), size of the oil futures market, global GDP, the number of US troops in the Middle East, and the frequency of fatal terrorist attacks in the Middle East are important driving forces. Turhan et al. [36] studied the long-run dynamic relationships between crude oil and stock markets, bond markets, gold, and the dollar index. They found that the correlation coefficient between crude oil and 10-year bond yield was hovering around zero before 2008 but after 2008, it is found to be around 0.25.

Crude Oil has a relationship with several other important financial instruments. Chen and Xu [37] developed a novel *Generalized Auto-Regressive Score* model to forecast the correlation between Crude Oil and Gold. Kumar [38] found non linear relationships between Crude Oil and Gold prices from an Indian perspective. We also studied in past the co-movement of Crude Oil and Gold prices with Indian stock index [39]. Wang and Chueh [40] found out that in the short term, Gold prices and Crude oil prices have a positive influence on each other; while in long term the interest rates have a negative influence on future Gold prices and a positive influence on future Crude Oil prices. They also found a long-run relationship, when interest rates influence the US dollar and in turn, the US dollar influences Crude Oil prices.Almost no relationships have been observed between Bitcoin and Crude Oil markets ([41–43]). Balcilar et al. [44] found a bidirectional return and volatility spillover for the Crude Oil, Gold, and S&P 500 index. Wu and Zhang [45] studied the role of China on Crude Oil prices. They did not find any significant role of the Chinese economy on Crude Oil prices, and on the contrary, they found that the contribution of Chinese economy is even less than the US Dollar Index effect on Crude Oil prices. But the research has been conducted on the data sets from October 2005 to November 2013. The Chinese economy has changed a lot since 2013. So, we need to again examine the effect of the Chinese economy on Crude Oil prices. Filippidis et al. [46] found out that the European Economic and Monetary Union (EMU) being the largest importer of Crude Oil, has an influence on Crude Oil prices. The correlation between 10 Year Yield spread and crude oil prices is time-varying which is again influenced during specific economic and geopolitical events. It is worth mentioning here that the spread is between the 10-year government bond yield issued by an EMU member country and the German Bund 10-year bond yield. The EMU countries are Austria, Belgium, Finland, France, Germany, Greece, Ireland, Italy, Netherlands, Portugal, and Spain.

## 3 Aims and objectives

Crude Oil is the most liquid commodity in Chicago Merchantile Exchange (CME) ([47–49]). Therefore, there is a huge number of options traders trading in the market. By *Options*, we mean *Call and Put options* and their various combinations. The options traders trade the volatility of the underlying, with the help of various strategies like the Butterfly strategy, Strangle strategy, etc ([50, 51]). As a result, the variance of Crude Oil and its forecasting is very important for the traders. Moreover, *options* are also very important for hedging purposes.

Recently, Urolagin et al. [49] forecasted the price of Crude Oil using Multivariate LSTM. They used the Gold Prices, S&P Index, Dollar Index, US 10 Year Bond yield to forecast the Crude Oil prices. Though they attained a whooping R-squared value of 95.4%, it comes at the cost of filtering the data to 19.25% of the original size for Mahalanobis distance, and 9.62% for Z-score based outlier detection. Mahalanobis distance, in short, can be defined as some metrics that measures the distance between a point and a distribution. In easy language, Z-score can be said as some metrics which can give us the knowledge about how much far a point is from the mean. In their own language “Even though the outlier elimination has reduced the size of the data set nominally, but there is a significant improvement in the performance of both these models.”. Now as an example, during the recent Oil crash on April 20, 2020; many investors earned huge money, while many lost their everything ([52–55]). So, keeping this in mind, we did not filter out the data and used the data as it is.

They also mentioned in their paper, “During preprocessing, the missing values for the attributes were adjusted by taking the previous day’s values. Since data are not in a normal distribution, therefore mean cannot be used to replace the missing values”. That is a major shortcoming in their paper because oil is the most volatile commodity. As an example, let us take three consecutive days. 17 April 2020, 20 April 2020, and 21 April 2020 (18 April 2020, and 19 April 2020 are Saturday and Sunday respectively). The closing prices of Crude Oil on 17, 20, 21 April were 18.27$, -37.63$, and 10.01$. Now if we are having missing values on 20 April and if we take the value of 17 April for the missing value of 20 April, we shall get the wrong interpretation.

In the present paper, we are influenced by Hui’s paper [28]. She used the dataset from 2006 to 2011. Similar to her findings, we tried to study the effect of inventory information shock on crude oil price variance. There is a small difference between our work and Hui. Hui studied the crude oil volatility while we studied the daily crude oil price variance.

In her research, she studied the inventory information shock and its effects on Crude Oil prices’ conditional variance with the help of the GARCH (1,1) method. In our research, we incorporate non-linear deep learning algorithms like LSTM for the first time to study the inventory information shock and its effects on Crude Oil price variance.

In this paper, we study the variance of Crude Oil prices and try to forecast it with the help of Multivariate LSTM methods. At first, we take the variances of Gold, US 10 Year Bond Yield, German 10 Year Bund Yield, Bitcoin, S&P 500 Index, Shanghai Stock Exchange Index, Dollar Index, and previous day Crude Oil prices; and the Inventory levels. We then try to verify the results of Inventory with Hui [28] and examine whether her results are still valid in these changing times? The various factors are selected based on their ability to Granger Cause Crude Oil variance. The selected factors are used to predict the variance of Crude Oil prices. Various hyperparameters of LSTM are tuned to get the best predicted results. In our research, we did not filter out extreme values as big profits and losses are made on that very days.

## 4 Methodology: Description of analysing tools

### 4.1 Data description

For this study, the data has been collected for the end-of-day Crude Oil price variance, 10-Year US Bond yield price variance, Bitcoin Price variance, S&PIndex variance, Gold Price variance, Shanghai Stock Exchange Index variance, Dollar Index variance, German Bund Yield variance, inventory levels, and inventory levels estimations. The data was collected from investing.com. The period of the data is from November 2010 to June 2020, on both a daily and weekly basis. The markets in which the instruments are traded along with their symbols are given in Table 1.

**Table 1:** Symbols of the financial instruments along with the markets they are traded in are shown.

Factors	Symbols	Markets in which it is traded
Bitcoin	BTC	Binance, Coinbase Pro. & OKEx.
S&P 500 Index	GSPC, INX and $SPX	NYSE, NASDAQ,and Cboe BZX Exchange
Gold	GC	New York Commodities Exchange (COMEX)
Shanghai Stock Exchange Composite Index	000001.SS	Shanghai Stock Exchange (SSE)
Dollar Index	USDX	Intercontinental Exchange (ICE)
10 Year German Bund Yield Futures	FGBL	European Derivatives Exchange (EUREX)
10 Year US Bond Yield Futures	US10Y	New York Stock Exchange (NYSE)

We take the rolling variance of the last ten days and try to forecast the variance of Crude Oil prices with the help of several other variances and the inventory factors. We have deliberately chosen the time frame from November 2010 to June 2020 to portray the events of the last decade.

Several economic incidents took place in the last decade. It all started with the earthquake in Japan causing damage of 18000 lives and property damage of 220 billion dollars [56]. In 2015 we saw the Greek debt crisis. The Greek economy almost became bankrupt [57]. It is said that the Greek rescue is the biggest rescue of any country in history. In the same year, based on the *purchasing power parity*, China became the first on the globe [58]. Moreover, China is also the second-largest holder of US debt [59]. In June 2017, United Kingdom voted to move out of European Union [60]. This act was popularly known as “Brexit”. Though it was assumed that it will hamper the economic growth of Britain but UK’s output rose to pre-Brexit times by 2020 [61]. On March 11, 2020, WHO declared COVID 19 a pandemic [62]. Thereafter, major economic downturns started to take place around the globe due to the spread of this virus. These are the major economic events that took place around the globe in the last decade.

#### 4.1.1 Inventory shock

*Inventory Shock* is a term being created by Anderson et al. [63] which is the difference between the real Inventory Changes and the expected Inventory Changes. It is given by

$$Invshock_{t}=\Delta Inv_{t-1}-E(\Delta Inv_{t-1})$$

Here,Δ*Inv*_*t*−1_ signifies the change of inventory levels in one week. The estimated values,E(Δ*Inv*_*t*−1_), and the original values, Δ*Inv*_*t*−1_, are taken from investing.com.

Inventory Information shock is the difference between actual inventory changes and expected inventory changes. As a matter of fact, every Tuesday we get an expected value of inventory change, and on Wednesday, at about 8 a.m. E.T. the actual inventory change is announced. The expected inventory change is given by Reuters and the actual inventory change is announced by the U.S. Energy Information Administration.

Hui [49] introduced several dummy variables such as *D*_*EIA*_, *D*_*EIA*,*pos*_*andD*_*EIA*,*neg*_ which were used to identify the days of inventory announcements, the days of positive inventory shocks, and the days of negative shocks respectively.*D*_*EIA*_|*Inv*_*EIA*,*tau*_| is the absolute shock term. The values related to inventory remained the same from one announcement to the next announcement.

### 4.2 Granger causality tests

We perform the Granger Causality test to examine whether a particular parametre has any effect on the variance of crude prices or not? We will choose only those parametre(s) for our analysis, which Granger cause Crude Oil price variance.

### 4.3 Long short-term memory

In 1997, Hochreiter and Schmidhuber [64] brought out a new method known as Long Short-Term Memory (LSTM) which solves complex, artificial long-time-lag tasks that have never been solved by previous recurrent network algorithms. LSTM has the inherent quality of remembering information for long periods. LSTM has a chain of repeating modules where each module has four neural networks.

**Fig 1:**
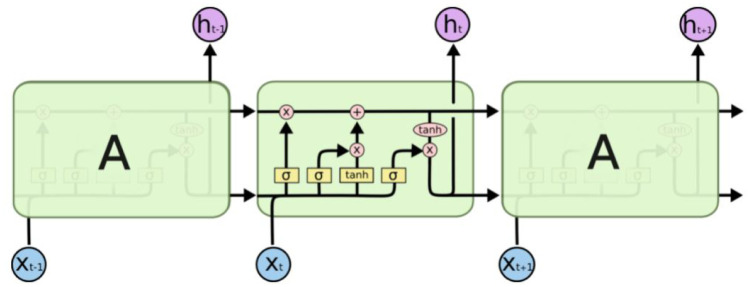
A sample LSTM unit. Credits: Christopher Olah [65].

In the Fig 1, we can see an LSTM structure. We can see the cell state at the top of the figure.

At the very beginning, a decision is taken on the retention rate of the information. For that, we introduce the concept of ‘forget gate’. It carries the information through each module with minor modifications. It concatenates the value of *h*_*t*−1_ and *x*_*t*_, outputs a value of 0 or 1 for each number of Cell State *C*_*t*−1_. A ‘1’ means 100% of the information is to be retained whereas a ‘0’ means 0% of the information is to be retained. The equation is given in Eq (1).

$$\begin{array}{c} f_{t}=\sigma \left(W_{f}.\left[h_{t-1},x_{t}\right]+b_{f}\right) \end{array}$$ (1)

Then we decide the information that is to be added to the cell state. This step has two layers viz. sigmoid layer and the tanh layer. The sigmoid layer will give the information on which values to be updated. The tanh layer gives a vector of values. The situation is given in Eqs 2 and 3.

$$\begin{array}{c} i_{t}=\sigma \left(W_{i}.\left[h_{t-1},x_{t}\right]+b_{i}\right) \end{array}$$ (2)

$$\begin{array}{c} X_{t}=tanh\left(W_{c}.\left[h_{t-1},x_{t}\right]+b_{c}\right) \end{array}$$ (3)

In Eq 3, the *X*_*t*_ that is calculated are to be added to the cell state. We then modify the old cell state to the new cell state. At first, we forget a part of the information from the old cell state and then add new information to the modified old cell state to obtain the new cell state. The situation is given in Eq 4.

$$\begin{array}{c} C_{t}=f_{t}*C_{t-1}+i_{t}*X_{t} \end{array}$$ (4)

The output depends on the modified cell state. Then we pass the output of the last module and the input of the present module through the sigmoid gate. On the other hand, the cell state is passed through the tanh layer to get the resultant between -1 and 1 and is multiplied by the output of the sigmoid gate. The equations are given below.

$$\begin{array}{c} o_{t}=\sigma \left(W_{o}\left[h_{t-1},x_{t}\right]+b_{0}\right) \end{array}$$ (5)

$$\begin{array}{c} h_{t}=o_{t}*tanh\left(C_{t}\right) \end{array}$$ (6)

After that, we will optimize the lookback period, the epoch and the batch size. An epoch is just an hyperparametre that is the number of times the learning algorithm will work through the entire data set. While the batch size is a hyperparametre which gives the number of samples to work with before updating the internal model parameters.

We will use the Mean Squared Error as the loss function and R-squared to measure the accuracy.

The mean squared error is given by equation.

$$\begin{array}{c} MSE=\frac{1}{n}*\sum_{n=1}^{\infty }{\left(y_{i}-\hat{y_{i}}\right)}^{2} \end{array}$$ (7)

The R-squarred is given by the equation.

$$\begin{array}{c} R^{2}=1-\frac{\sum (y_{i}^{2}-{\hat{y_{i}}}^{2})}{\sum (y_{i}^{2}-{\bar{y_{i}}}^{2})} \end{array}$$ (8)

where *y*_*i*_ is the value of y at *i*^*th*^ point, $\hat{y_{i}}$ is the predicted value of y and $\bar{y_{i}}$ is the mean value of y.

### 4.4 Optimization

**Fig 2:**
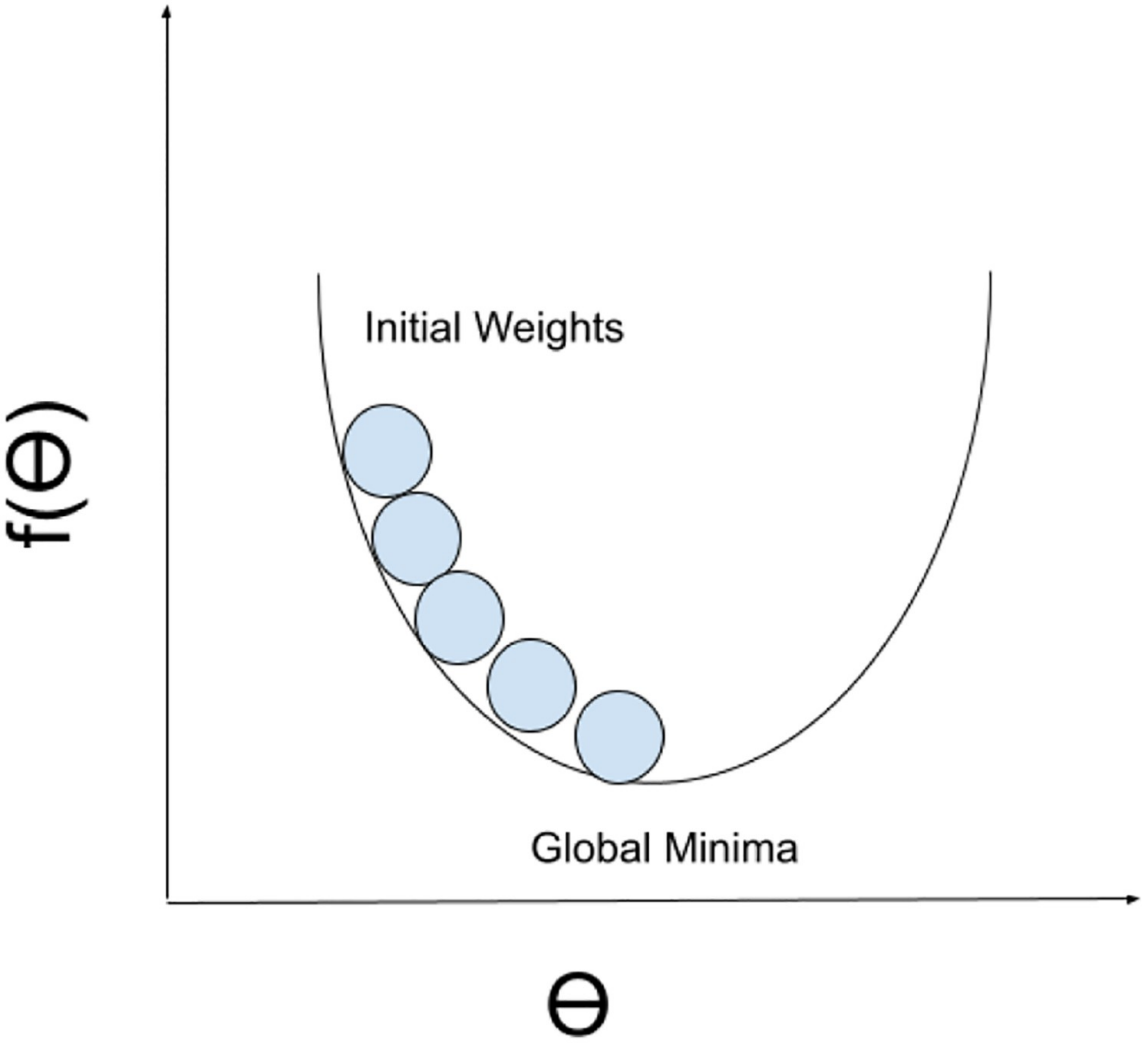
Gradient descent optimization. The figure shows incremental steps on how the algorithm comes to a point of minimum error.

We need to minimize the objective function f(*θ*) having parameters *θ*, $\eta \in R_{d}$. In doing so, we have to update the parameters in the opposite direction of the gradient of the objective function, ∇_*θ*_*f*(*θ*) with respect to the parameters *θ* and *η*. The parameter *η* decides the size of steps to be taken to reach local minima, and is called the learning rate. The whole process is known as Gradient Descent. The situation is shown in the Fig 2.

#### 4.4.1 Gradient descent optimization algorithms

We compare the two Gradient Descent Optimization Algorithms namely: *Adam* and *Nadam*. In a nutshell, these are discussed below:

#### 4.4.2 Adaptive moment estimation(Adam)

*Adam* computes the adaptive learning rates for each parameter associated with the objective function [66]. *Adam* keeps into account the exponentially decaying average of past squared gradients *v*_*t*_ along with the exponentially decaying average of past gradients *m*_*t*_. These are computed as follows.

$$\begin{array}{c} m_{t}=\beta_{1}m_{t-1}+\left(1-\beta_{1}\right)g_{t} \end{array}$$ (9)

$$\begin{array}{c} v_{t}=\beta_{2}v_{t-1}+\left(1-\beta_{2}\right)g_{t}^{2} \end{array}$$ (10)

Here *m*_*t*_ and *v*_*t*_ are basically the mean and the uncentered variance of the gradients respectively. The parameters are then updated by the following equation.

$$\begin{array}{c} \theta_{t+1}=\theta_{t}-\frac{\eta }{\sqrt{\hat{v_{t}}}+\epsilon }\hat{m_{t}} \end{array}$$ (11)

#### 4.4.3 Nadam

*Nadam* is a combination of *Adam*, and Nesterov accelerated gradient (NAG) [67]. In combining so, we just modify the way we deal with the term *m*_*t*_ in *Adam*. In this case of *Adam*, to update *m*_*t*_ we use *m*_*t*−1_. But in *Nadam*, to update *m*_*t*_, we use the current *m*_*t*_ and not *m*_*t*−1_. The equation of parameter update is given below.

$$\begin{array}{c} \theta_{t+1}=\theta_{t}-\frac{\eta }{\sqrt{\hat{v_{t}}}+\epsilon }\left(\beta_{1}\hat{m_{t}}+\frac{\left(1-\beta_{1}\right)g_{t}}{1-\beta_{1}^{t}}\right) \end{array}$$ (12)

### 4.5 Shapley values

All the features of the model do not have the same amount of influence on prediction. To study this, the features can be examined individually. But again, this method won’t allow us to study the interdependence between features. To study the interdependence between features, we study the influence of each possible set of different features on prediction separately. These changes are then combined to present the contributions we get from each feature. That is, feature values work together in a model to change its predictive power with respect to the model’s output, and the Shapley values divide this total change in prediction among the features so that the process is fair among all the subsets of features.

To explain the concept of Shapley values [68, 69], we have to define and explain some terms. First and foremost, we have to define the concept of Cooperative game theory.

#### 4.5.1 Cooperative game

Let {1, 2, 3, …, *p*} be a finite set of players. Let $\nu :2^{p}\to R$ be a characteristic function, where we are having *ν*(*ϕ*) = 0, and let ({1, 2, 3, …, *p*}, *ν*) be a tuple. Any subset *S*, *S* ⊂ {1, 2, 3, …, *p*}, is called a coalition and the set {1, 2, 3, …, *p*} consisting of all the players is called a grand coalition. The characteristic function *ν* gives an account of the worth of each coalition. Thereby, we can form the grand coalition, and split the worth among the players in a most **fair** way. As a solution, we get a vector *ϕ* = (*ϕ*_1_, *ϕ*_2_, …., *ϕ*_*p*_). It is nothing but a vector of payoffs for the game ({1, 2, 3, …, *p*}, *ν*).

There are four axioms which take the responsibility of fairness for a game. These are listed below.

1. Efficiency: It ensures the following equality.
  $$\begin{array}{c} \phi_{1}\left(\nu \right)+\phi_{2}\left(\nu \right)+\ldots .+\phi_{p}\left(\nu \right)=\nu \left(\left\{1,\ldots ,p\right\}\right) \end{array}$$ (13)
2. Symmetry: If for every set S, *S* ⊂ {1, …*p*}, there exist two players; i and j; such that *i*, *j* ∉ *S*, and the following equality holds, *ν*(*S* ∪ {*i*}) = *ν*(*S* ∪ {*j*}), then the following equality *ϕ*_*i*_(*ν*) = *ϕ*_*j*_(*ν*) is satisfied.
3. Dummy: For every set *S*, where *S* ⊂ {1, …, *p*}, *i* ∉ *S* and *ν*(*S* ∪ {*i*}) = *ν*(*S*), we have *ϕ*_*i*_(*ν*) = 0.
4. Additivity: It ensures that for any pair of games, where *ν*, *w*;(*ν*+ *w*)*S* = *ν*(*S*) + *w*(*S*), we have the equality *ϕ*(*ν* + *w*) = *ϕ*(*ν*) + *ϕ*(*w*).

The **Shapley Value** is used to distribute the total gains to the p players in an impartial way. The total gains is defined as v(1, …, p). Shapley Value ensures that the above four axioms are satisfied.

The **Shapley Value** can be defined as the unique solution *ϕ* which satisfies the axioms from 1 to 4 for the game ({1, …, *p*}, *v*). Mathematically, it is given as:

$$\begin{array}{c} Sh_{i}\left(\nu \right)=\Sigma_{S\subseteq \{1,\ldots ,p\}-\{i\}}\frac{(p-|S|-1)!|S|!}{p!}\left(\nu \left(S\cup \left\{i\right\}\right)-\nu \left(s\right)\right); \end{array}$$ (14)

*i* = 1, …, *p*

*4.5.1.1 Method for computing Shapley Values.* We are having *X* = *X*_1_ × *X*_2_ × *X*_3_ × …. × *X*_*p*_ as the feature space of *p* features, with *f* being the model that is to be explained. Here, let us take *y* = (*y*_1_, *y*_2_, *y*_3_, …., *y*_*p*_) ∈ *X* be any instance from the feature space. We want to explain the prediction of *y*. Now as per Strumbelj and Kononenko, **Prediction Difference** of a subset of feature values, gives us the change in expectation caused by those feature values [70]. Mathematically, **Prediction Difference** is given as:

If *f* is a model and *S* = {*i*_1_, *i*_2_, *i*_3_, …., *i*_*p*_} ⊆ {1, 2, …, *p*} is a subset of features, then the prediction difference Δ^*y*^(*S*) in instance *y* = (*y*_1_, *y*_2_, …., *y*_*p*_) ∈ *X*, for a subset of features *S* ⊆ {1, 2, 3, …, *p*} is defined as,

$$\begin{array}{c} \Delta^{y}\left(S\right)=E\left[f\left(X_{1},\ldots .,X_{p}\right)|X_{i_{1}}=y_{i_{1}},\ldots .,X_{i_{n}}=y_{i_{n}}\right]-E\left[f\left(X_{1},\ldots .,X_{p}\right)\right] \end{array}$$ (15)

Here we can see that, Δ^*y*^ is a function from the set of all possible subsets of the features to R, i.e., $\Delta^{y}:2^{p}\to R$, which satisfies the condition Δ^*y*^(*ϕ*) = *E*[*f*(*X*_1_, …, *X*_*p*_)] − *E*[*f*(*X*_1_, …, *X*_*p*_)] = 0. Therefore, it can be said that Δ^*y*^ is a characteristic function for the cooperative game consisting of *p* players. In this case, the worth of coalitions, is the change in the model’s prediction given by Δ^*y*^. Our aim is to split the total **Prediction Difference**, Δ^*y*^({1, …, *p*}) among all the features in an impartial way. For that **Shapley Value**, of the cooperative game represented by ({1, …, *p*}, Δ^*y*^), comes to our rescue. To compute the contribution of the *i*^*th*^ feature in the prediction of y, we have the following formula for **Shapley Value** of *i*^*th*^ as:

$$\begin{array}{c} \phi_{i}\left(\Delta^{y}\right)≔Sh_{i}\left(\Delta^{y}\right)=\Sigma_{S\subseteq \{1,\ldots ,p\}-\{i\}}\frac{(p-|S|-1)!|S|!}{p!}\left(\Delta^{y}\left(S\cup \left\{i\right\}\right)-\Delta^{y}\left(S\right)\right) \end{array}$$ (16),

*i* = 1, …, *p*. We will be now examining whether the three axioms described earlier, are satisfied by **Shapley Value** or not?

1. Efficiency Axiom: Here, we have the sum of contributions from the *p* features and it is equal to the difference between the prediction of the model for the instance and the model’s expected output. In this situation, we are not given any information about the instance’s feature values. The mathematical equation is given below.
  $$\begin{array}{c} \phi_{1}\left(\Delta^{y}\right)+\phi_{2}\left(\Delta^{y}\right)+\ldots .+\phi_{p}\left(\Delta^{y}\right)=E\left[f|y\right]-E\left[f\right]=f\left(y\right)-E\left[f\right] \end{array}$$ (17)
2. Symmetry Axiom: Identical contributions are assigned for two features having identical influence on the predictive capacity on the model. Mathematically, if there are two players *i* and *j*, satisfying the condition Δ^*y*^(*S* ∪ {*i*}) = Δ^*y*^(*S* ∪ {*j*});*S* ⊂ {1, …, *p*} and *i*, *j* ∉ *S*, then the following equality is satisfied.
  $$\begin{array}{c} \phi_{i}\left(\Delta^{y}\right)=\phi_{j}\left(\Delta^{y}\right) \end{array}$$ (18)
3. A feature which has no influence on the prediction of the model, has a contribution of 0. Mathematically, for every set *S*, *S* ⊂ {1, 2, …, *p*} and *i* ∉ *S*; we are having
  $$\begin{array}{c} \phi_{i}\left(\Delta^{y}\right)=0. \end{array}$$ (19)

The pseudocode for Shapley Value is given below:

Initialize Model *f*, instance *y*, and number of samples *N*

*ϕ*_*i*_ = 0

for j in range(1, N):

 $O\varepsilon S_{p}$ is chosen as a random permutation of features

 A random instance z∈X

 if $k\in Pre^{i}\left(O\right)\cup \left\{i\right\}:$

  $x_{k}^{\prime }=y_{k}$

 else:

  $x_{k}^{\prime }=z_{k}$

 if $k\in Pre^{i}\left(O\right)$:

  $x{\prime \prime }_{k}=y_{k}$

 else:

  $x{\prime \prime }_{k}=z_{k}$

  $\phi_{i}=\phi_{i}+f\left(x^{\prime }\right)-f\left(x\prime \prime \right)$

 $\phi_{i}=\frac{\phi_{i}}{N}$

Here, $Pre^{i}O$ is the set of players which are predecessors of player *i* in permutation $O\varepsilon S_{p}$. In the above algorithm, we compute the *i*^*th*^ feature’s contribution (*ϕ*_*i*_) for model *f* and for instance y∈X. Here, *x*′ and x″ are two observations which differ only in the value of *i*^*th*^ feature. To construct *x*′ and x″, we take the instance *z*, and then change the value of each feature that appears before the *i*^*th*^ feature in the random permutation of features, O, to that feature’s value in *y*.

## 5 Results and discussions

In this research, we begin the process with the collection of data from investing.com. The data collected is for the period December 7, 2010, to June 9, 2020. Thus the data covers all important events like the Greek crisis, Brexit, Covid meltdown, etc. The inventory data is changed once every week. The actual inventory and the shock changes on Wednesday. After it is changed, the values remains the same for the whole week. The forecasted inventory value changes on Tuesday, and it remains the same for the whole week. We studied the effects of inventory on the variance of crude oil prices. This study will benefit the options trader who wants to benefit from the volatility of crude oil prices on the days of inventory announcements. We then tried to forecast the crude oil daily price variance.

We studied the granger causality of the different factors on the crude oil variance. We did not take the actual inventory as it has a correlation of above 0.5 with forecasted crude oil.

The factors which satisfy the granger causality tests are then taken into account for LSTM tests. In the LSTM tests, we optimized the lookback period and the gradient descent optimizer. The lookback period taken for studying is from 1 to 10 days. These 10 days will take care of 2 weeks on average. We did not take longer time periods as we have seen from Granger causality tests that variances of Gold and Dollar index influence even after 8 to 9 days. And the factors which do not influence crude oil daily variance even after ten days, are not taken seriously even if they show causality after 10 days. This is because it may be due to some spurious relationships. There may be some relationships between them in a weekly or monthly time frame, but in a fast-moving time frame, we neglect the relationships between these instruments after 10 days.

**Fig 3:**
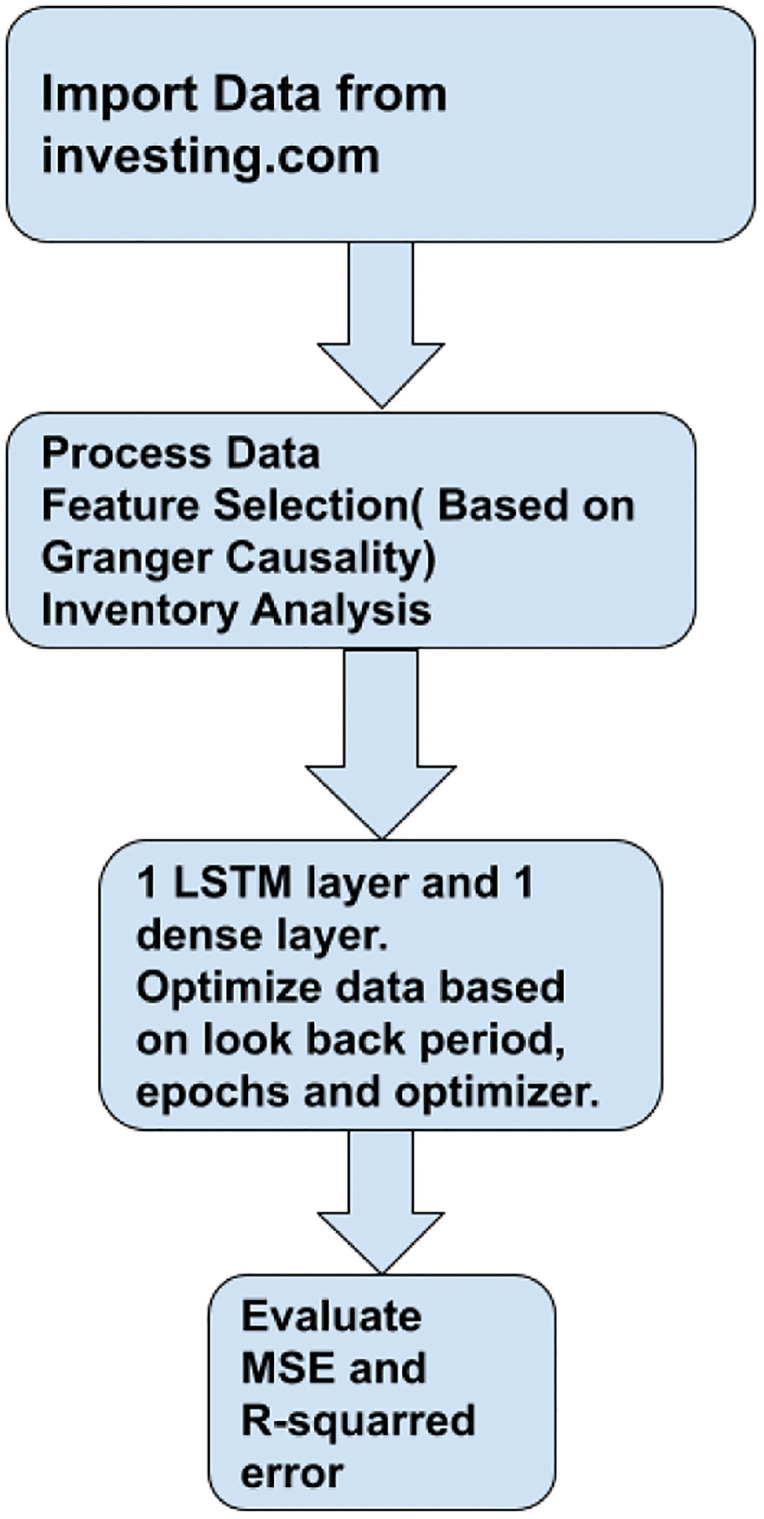
Flowchart of our method.

The LSTM and its optimization are then performed. The various statistical information of the factors is shown in the Fig 3. The various statistical information of the factors is shown in the Table 2. Except for Crude Oil Daily price volatility, other factors have 2218 data points from December 2010 to June 2020.

**Table 2:** Statistical Description of all the factors involved.

Factors	count	mean	std	min	25 perc.	50 perc.	75 perc.	max
Forecasted Inventory (scaled values)	2218	0.2075	2.417	-5.325	-1.700	0.238	1.967	15.150
BTC-Variance	2218	89325	365115	0	8.598	240	25778	5.524
S&P 500 Variance	2218	923	3515	7.596	117.64	269	623	60079
Gold Variance	2218	568	930	5	114	259	633	9128
Shanghai Stock Exchange Variance	2218	3639	10922	43	497	1045	2387	167244
Dollar Index Variance	2218	0.287	0.432	0.004	0.087	0.165	0.333	7.385
10-Year German Bund Yield Variance	2218	0.615	0.976	0.014	0.175	0.337	0.662	16
10-Year US Bond Yield Variance	2218	0.004	0.005	0.000043	0.001	0.002	0.004	0.069
Crude Oil Daily Price Variance	2209	5	21.98	0.079	1.044	1.971	4.032	372.298

### 5.1 Inventory information shock

In the story of Crude Oil for the last decade (2011-2020), inventory plays a major role. We verified whether anything changed in the last decade compared to the previous decade (2006-2011) as found out by Hui [28]. To begin with, Hui compared the EIA reports with the Reuters’ value. Hui found out that the correlation between them was 0.4412 whereas in our case we found it to be 0.5871. Hui had several concerns over the Reuters’ data. She mentioned in her paper, “We have two main concerns regarding Reuters’ data. First, although these data have been used, we prefer to provide some direct evidence of their accuracy. Second, there is the possibility that Reuters’ forecasts may not capture all information available immediately before the EIA announcement. New information may reach the market between the time the survey is conducted and the time when the actual value is realized, and accordingly, such information may alter the expectations…..”

Hui thereafter regressed the actual inventory announcements with forecasts and the changes of crude oil prices between the time of the survey and the time of the EIA announcements. This was done in order to verify the reliability of the survey. She had two equations. We verified one of the equations. The two equations are given below.

$$\begin{array}{c} \Delta Inv_{EIA,\tau }=\beta_{0}+\beta_{1}E{\left(\Delta Inv\right)}_{Reuters,\tau }+\beta_{2}\Delta P_{t}+e_{i\tau } \end{array}$$ (20)

$$\begin{array}{c} \Delta Inv_{EIA,\tau }=\beta_{0}+\beta_{1}E{\left(\Delta Inv\right)}_{Reuters,\tau }+e_{i\tau } \end{array}$$ (21)

**Fig 4:**
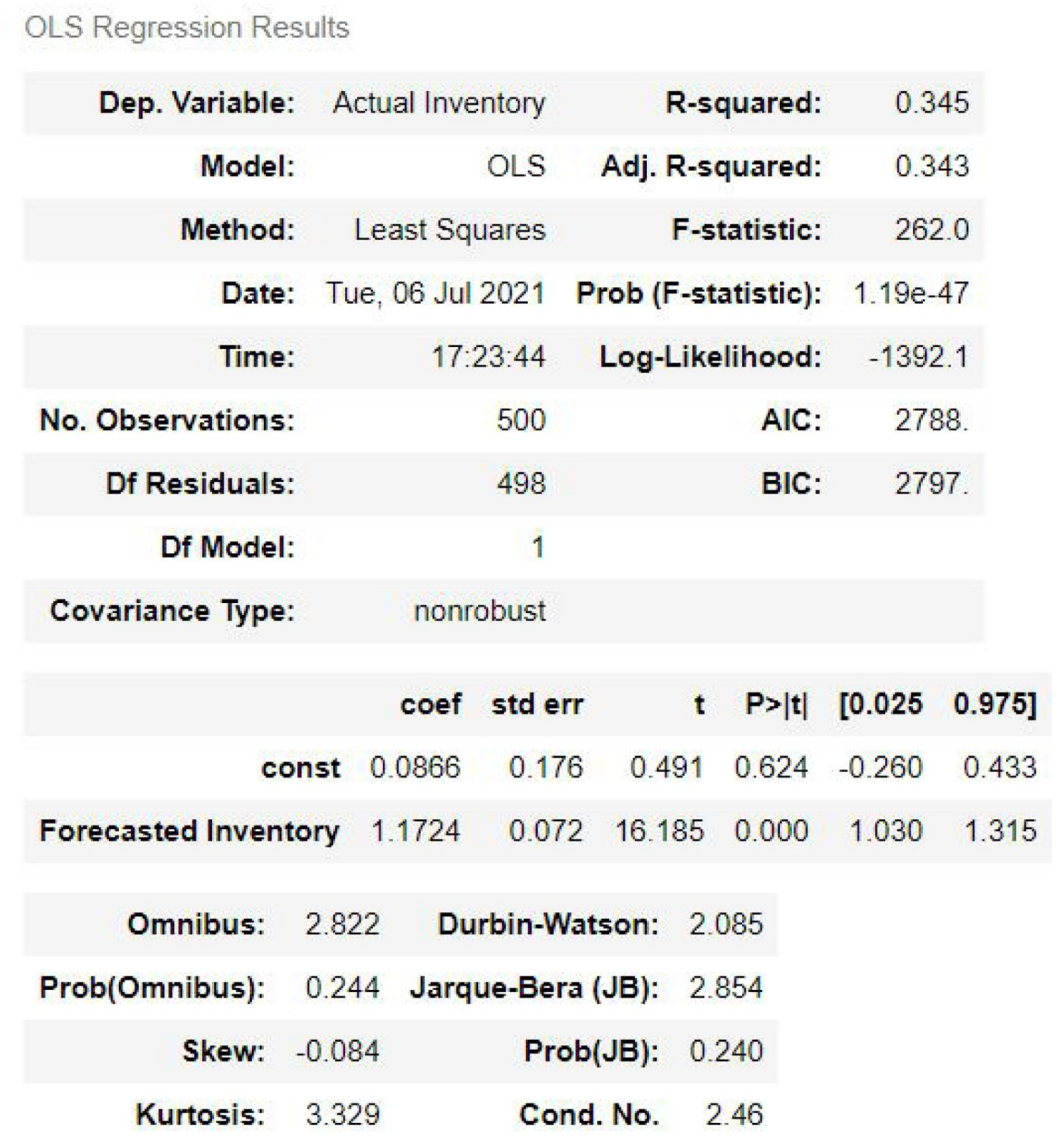
OLS regression results.

We reverified the second equation with the data for this decade (2011-2020). This is shown in Fig 4. By comparing the results for the period (2006-2011) with Hui [28], we see that their results are quite comparable with ours (2011-2020). Her Adjusted R-squared is 0.1926 whereas ours is about 0.343. Her DW test result is 1.9589; whereas ours is 2.085. This implies that nothing, as such, is changed in this time. On the contrary, we can say that we have got better results over the course of time.

In Table 3 we present the correlation between the inventory factors and daily Crude Oil price variance.

**Table 3:** Correlation between one of the inventory factors and crude oil price variance.

Factors	Correlation with Crude Oil price Variance
Forecasted Inventory	0.3705
Actual Inventory	0.2045
*D*_*EIA*_	-0.0009
*D*_*EIA*_|*Inv*_*EIA*,*tau*_|	-0.0186

Hui [28] mentioned in her paper, “To better understand the empirical results, we substitute actual inventory changes for inventory information shocks into the GARCH model. To do so, we incorporate *D*_*EIA*_*Inv*_*EIA*,*tau*_ into the mean equation and *D*_*EIA*_|*Inv*_*EIA*,*tau*_| into the conditional variance equation. We then compare the results of the models with inventory information shocks and with actual inventory changes to identify which part of the inventory information has the greatest impact on crude oil futures daily returns and volatility……”. She also mentioned “…while inventory shocks can significantly affect the mean of returns, such shocks do not significantly affect conditional variance…..”. As seen from our correlation and regression Tables 3 and 4, the conditional variance of daily Crude Price does not depend on Inventory Shock. The only difference between us and Hui is that she used conditional variance of daily returns while we used conditional variance of daily crude oil prices.

**Table 4:** Regression values of crude oil variance with forecasted inventory, actual inventory, and absolute shock.

Factors	Coefficient	std err	t	*P* ≥ |*t*|
const	3.9229	0.411	9.547	0.000
Actual Inventory	-0.0954	0.120	-0.794	0.428
Forecasted Inventory	3.3026	0.221	14.967	0.000
Absolute Shock	-0.1810	0.255	-0.710	0.478

**Fig 5:**
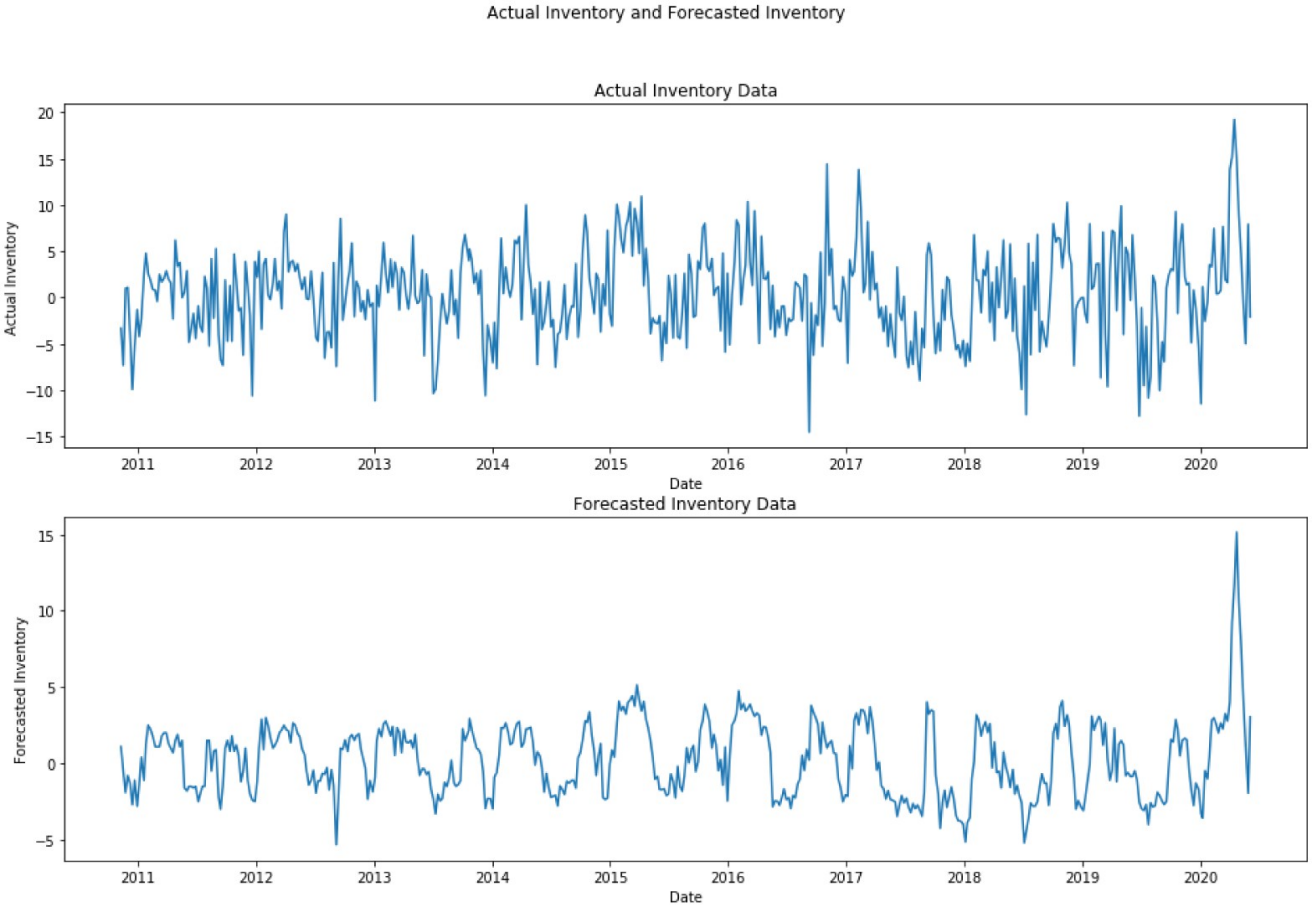
This diagram shows actual inventory and forecasted inventory on a weekly basis from 2011 to 2020.

So we could see that forecasted inventory and actual inventory are the major contributing factors for the variance of Crude Oil prices. But the correlation coefficient between forecasted inventory and actual inventory is 0.6057. So in order to avoid serial correlation, we only take forecasted inventory as one of the independent variables for forecasting crude oil variances. A diagram comparing actual inventory and forecasted inventory is given in Fig 5.

### 5.2 Granger causality tests

We performed the Granger Causality test for a lag term of 10 days, and we see that the forecasted inventory effects the crude oil price variance. So we have to accept our alternative hypothesis and claim that they are related.

Next we examine whether any of the following factors Bitcoin variance, S&P Index variance, Gold Price variance, Shanghai Stock Exchange Index variance, Dollar Index variance, German Bund Yield variance Granger cause crude oil price (variance) for a lag of 10 days or not?

Our results suggest that Bitcoin variance, Shanghai Stock Exchange variance, German 10-Year Bund Yield variance, US 10-Year Bond Yield variance do not Granger cause Crude Oil variance.

But the forecasted crude oil inventory, S&P Index variance and previous day Crude Oil Price variance Granger cause Crude Oil Price variance. Gold Price variance Granger causes Crude Oil variance after five days; while Dollar Index variance Granger causes Crude Oil variance after nine days.

So we choose forecasted crude oil inventory, S&P Index variance, Gold Price variance, and Dollar Index variance to be used as independent variable in order to predict Crude Oil price variance.

**Fig 6:**
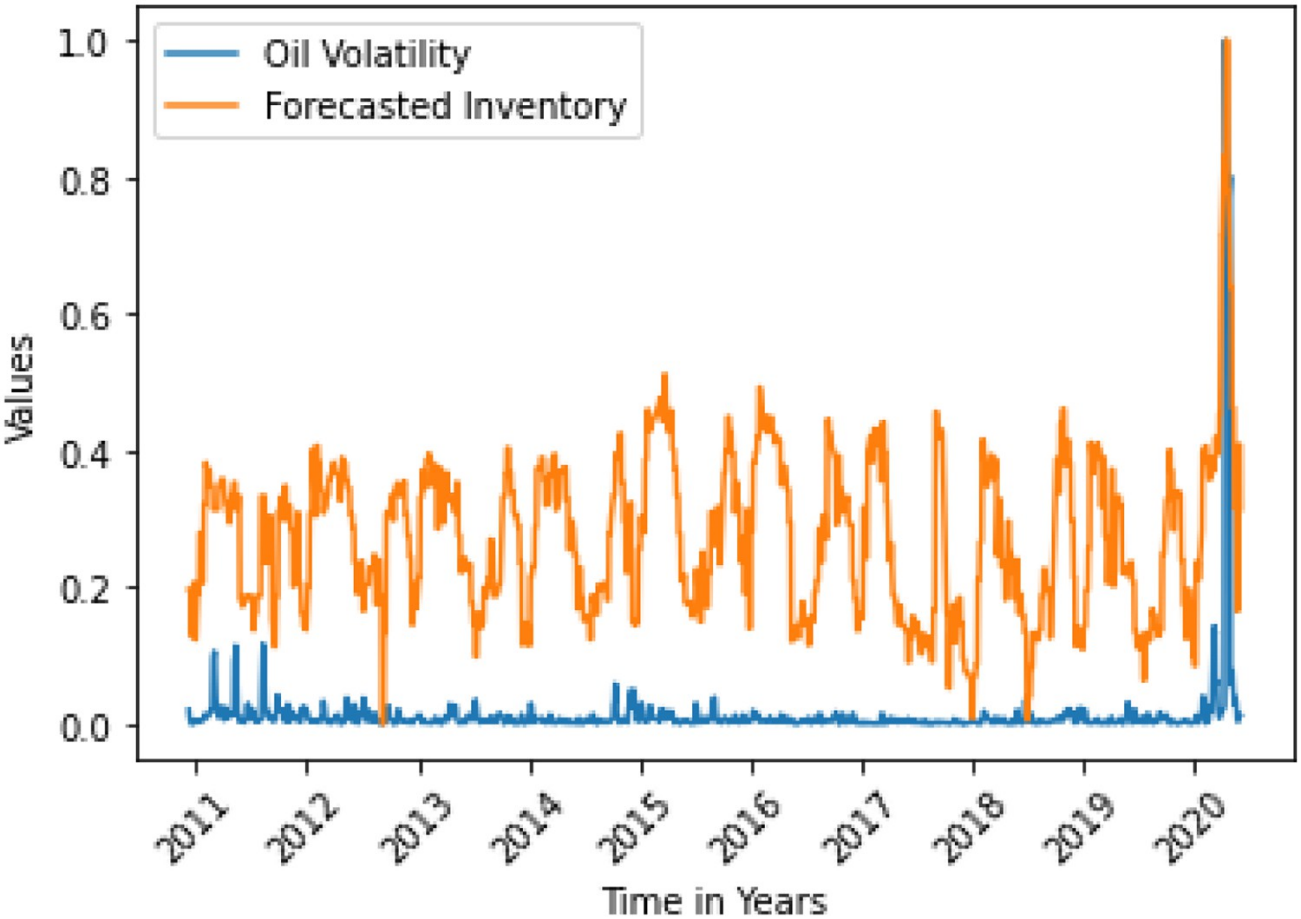
Forecasted inventory granger causes oil variance. Here, the correlation between two data is 0.3785.

The situations are shown in Figs 6 and 13.

**Fig 7:**
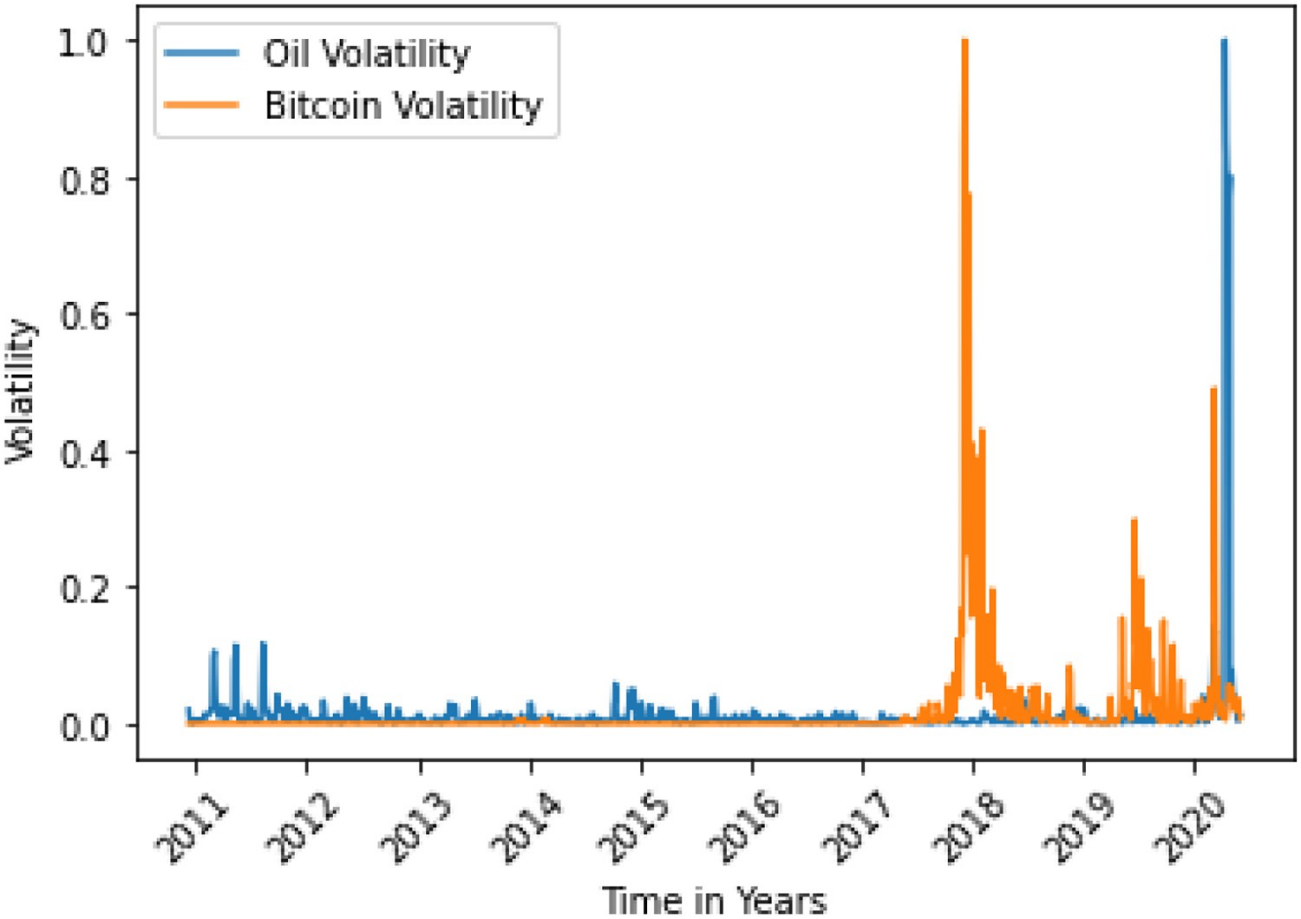
Bitcoin variance does not Granger cause oil variance. Here, the correlation between two data is 0.0188.

**Fig 8:**
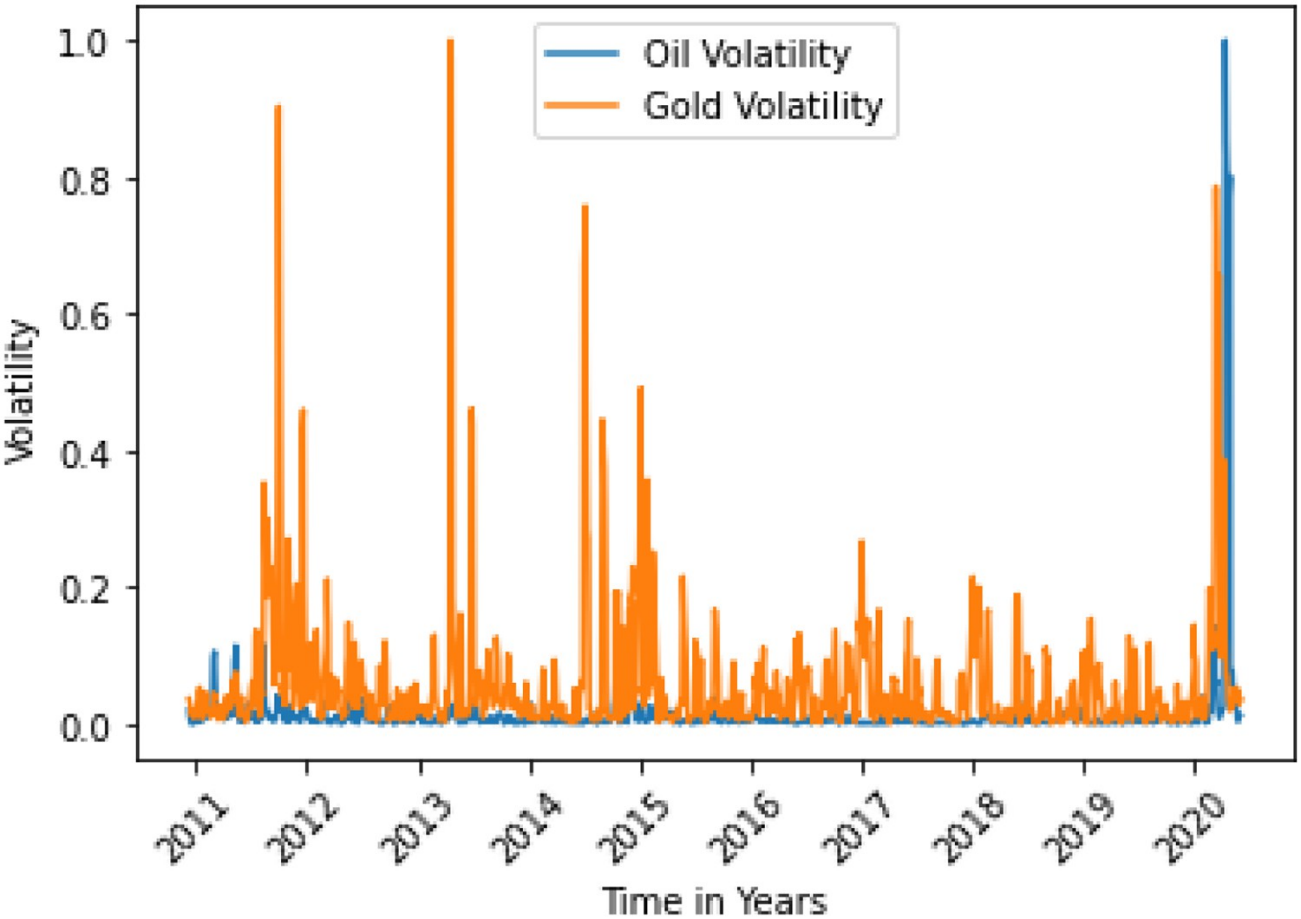
Gold variance Granger causes oil variance after 6 lags. Here, the correlation between two data is 0.0653.

**Fig 9:**
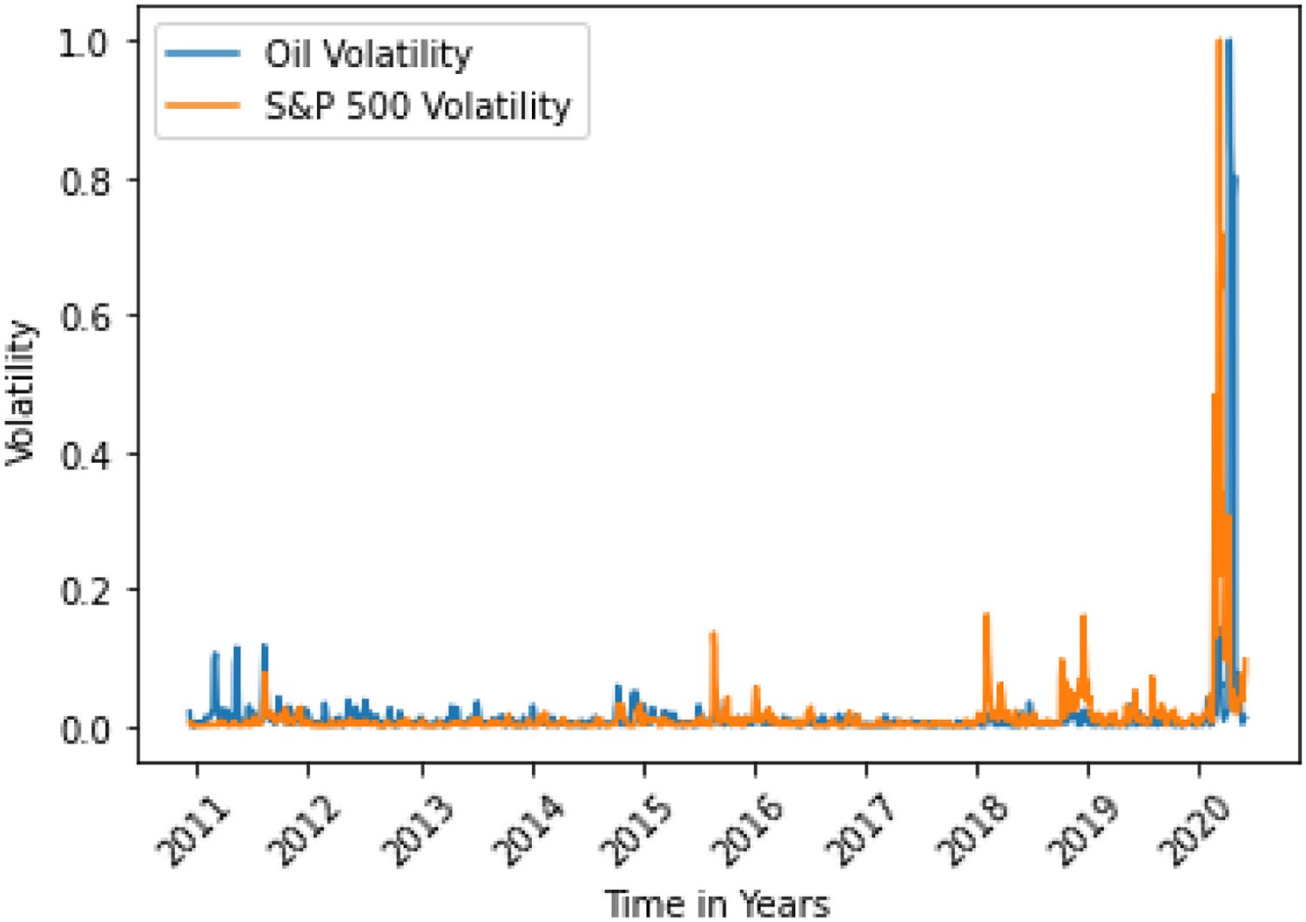
S&P 500 variance Granger causes oil variance. Here, the correlation between two data is 0.1589.

**Fig 10:**
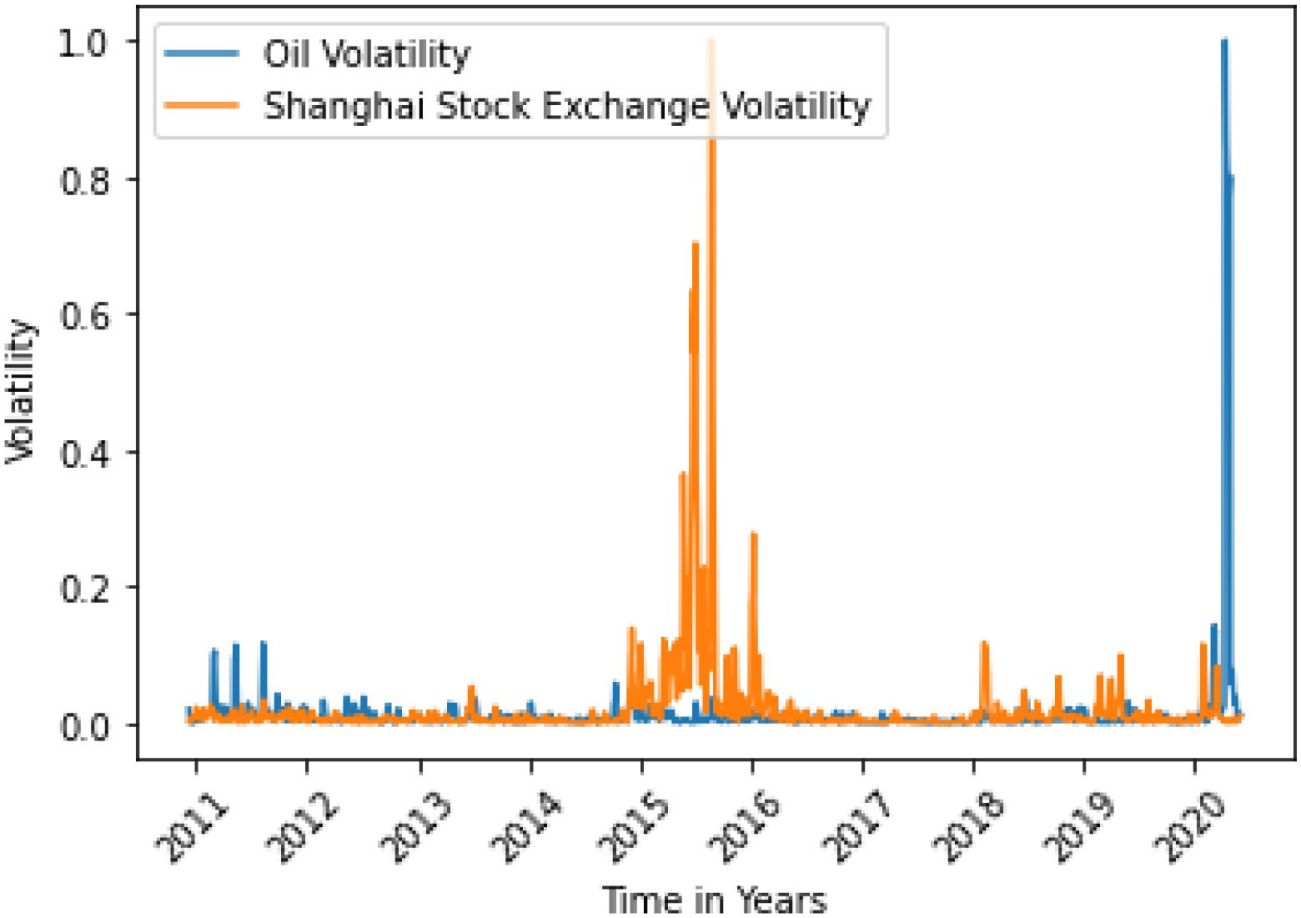
Shanghai Stock Exchange variance does not Granger cause oil variance. Here, the correlation between two data is -0.0019.

**Fig 11:**
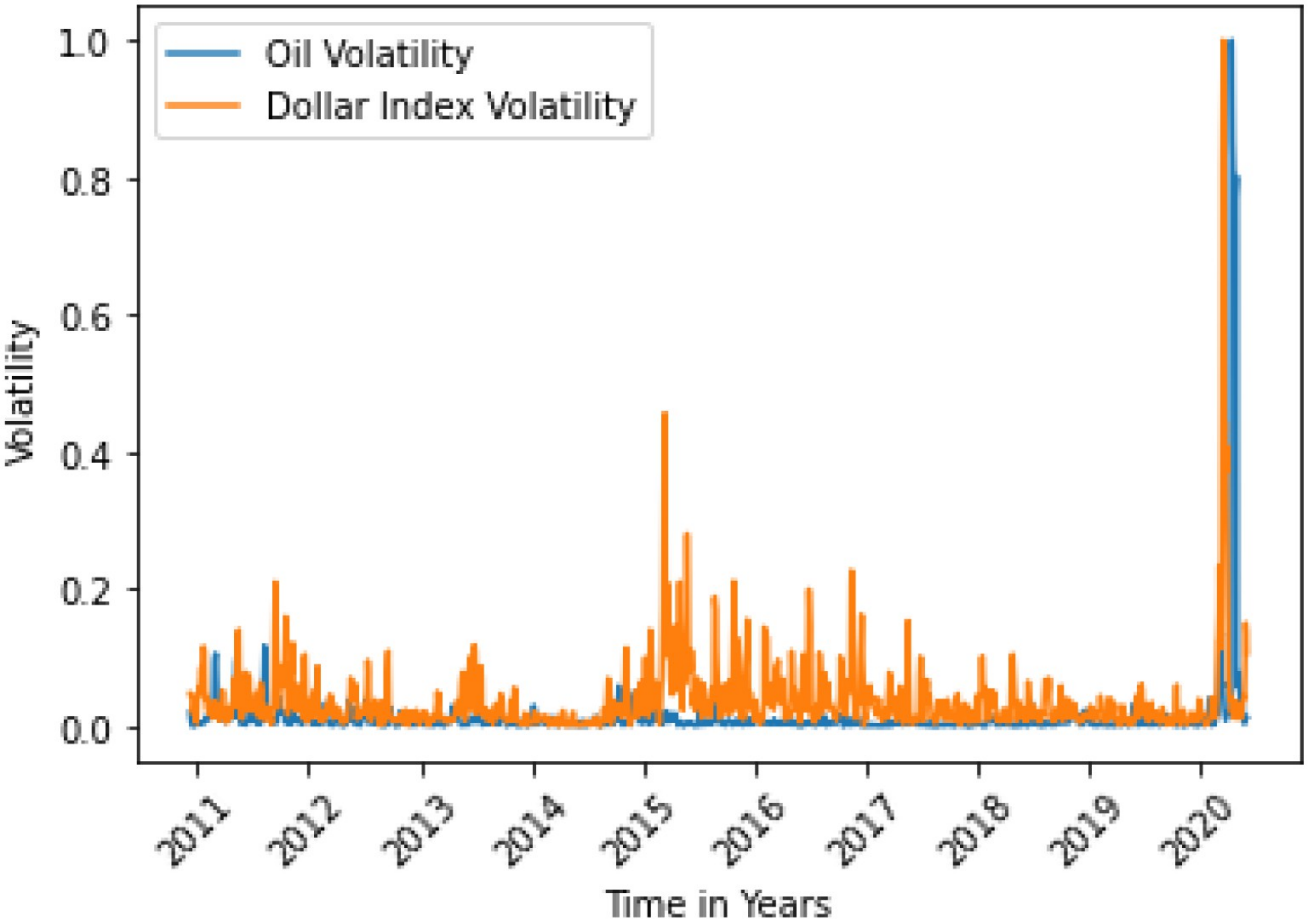
Dollar Index variance Granger causes oil variance after 7 days. Here, the correlation between two data is 0.0388.

**Fig 12:**
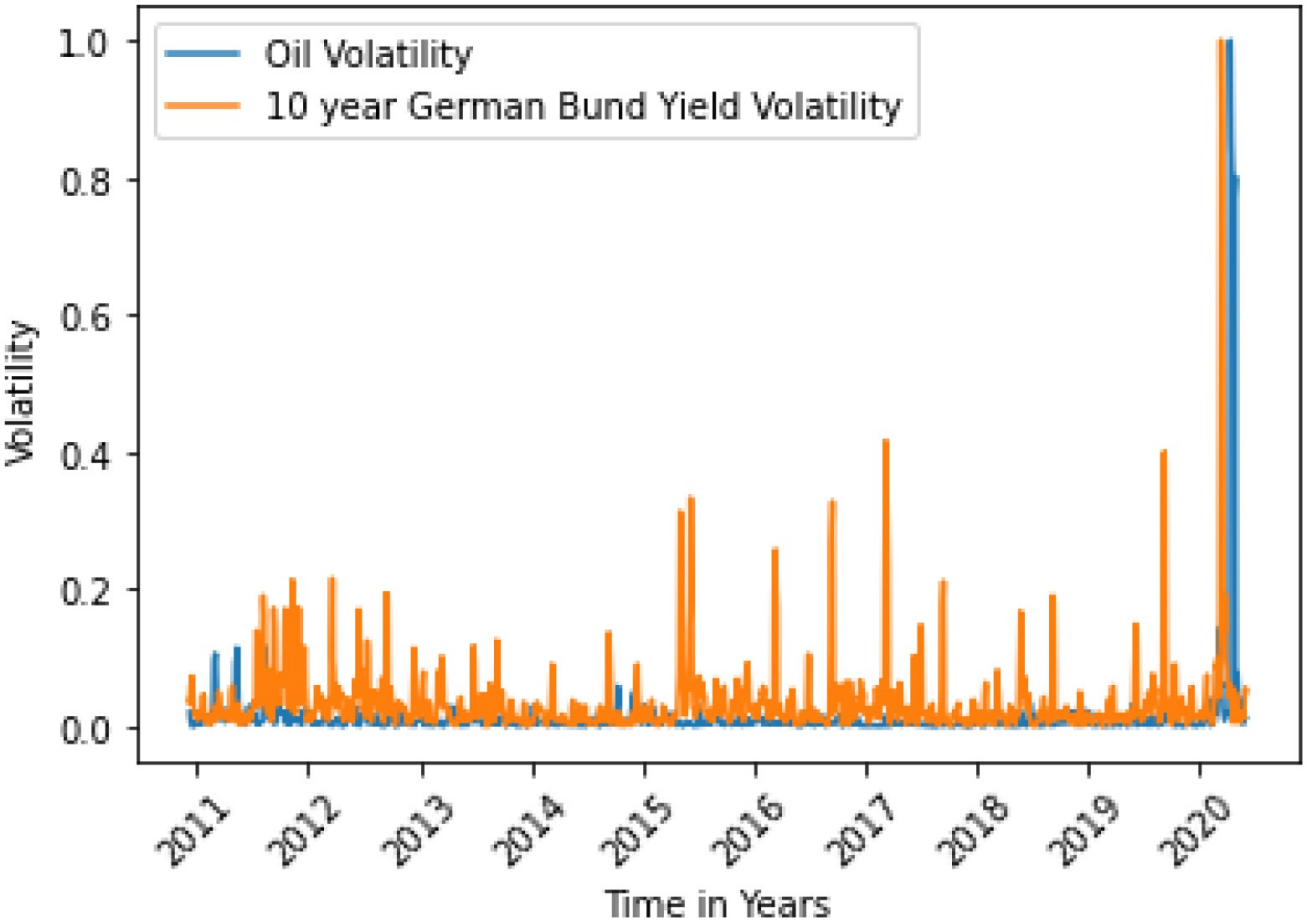
German 10 yr Bund Yield variance does not Granger cause oil variance. Here, the correlation between two data is 0.0433.

**Fig 13:**
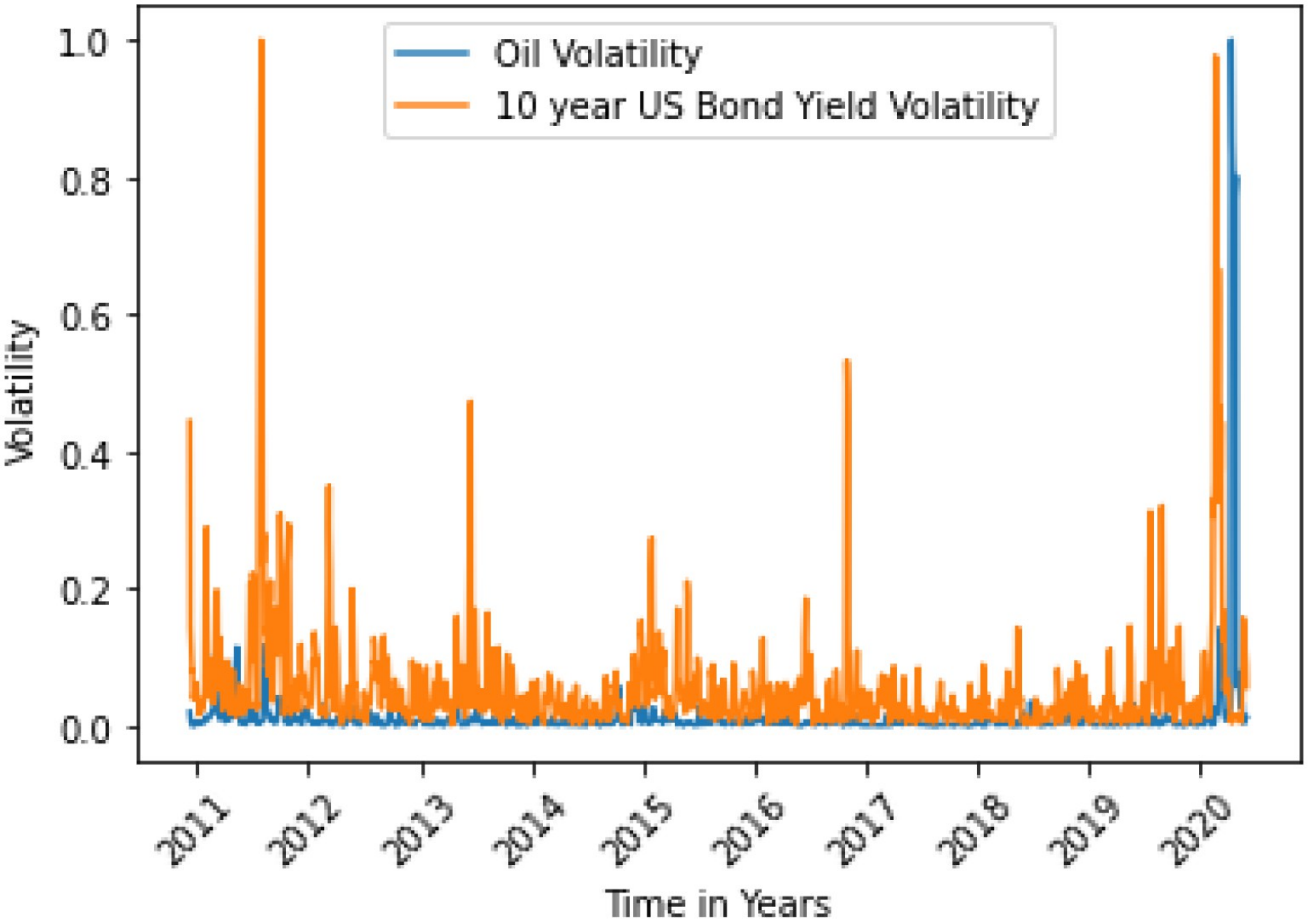
US 10 yr Bond Yield variance does not Granger cause oil variance. Here, the correlation between two data is 0.0241.

### 5.3 Long Short-Term Memory (LSTM) test results

Crude Oil is the most volatile commodity traded in the market. As such, forecasting the variance of crude oil prices is one of the toughest and most important works. After performing the Granger causality tests, we are left with four variables. These are 1. Forecasted inventory, 2. S&P 500 index daily variance, 3. Gold daily price variance, and 4. Dollar index daily variance. We also used the previous day’s crude oil price variance as one of the deciding factors to forecast crude oil variances. One of the most important aspects of our research as compared to Urolagin et al. [49] is that we did not filter out the extreme values as those extreme values are very important for wealth creation and wealth destruction.

**Fig 14:**
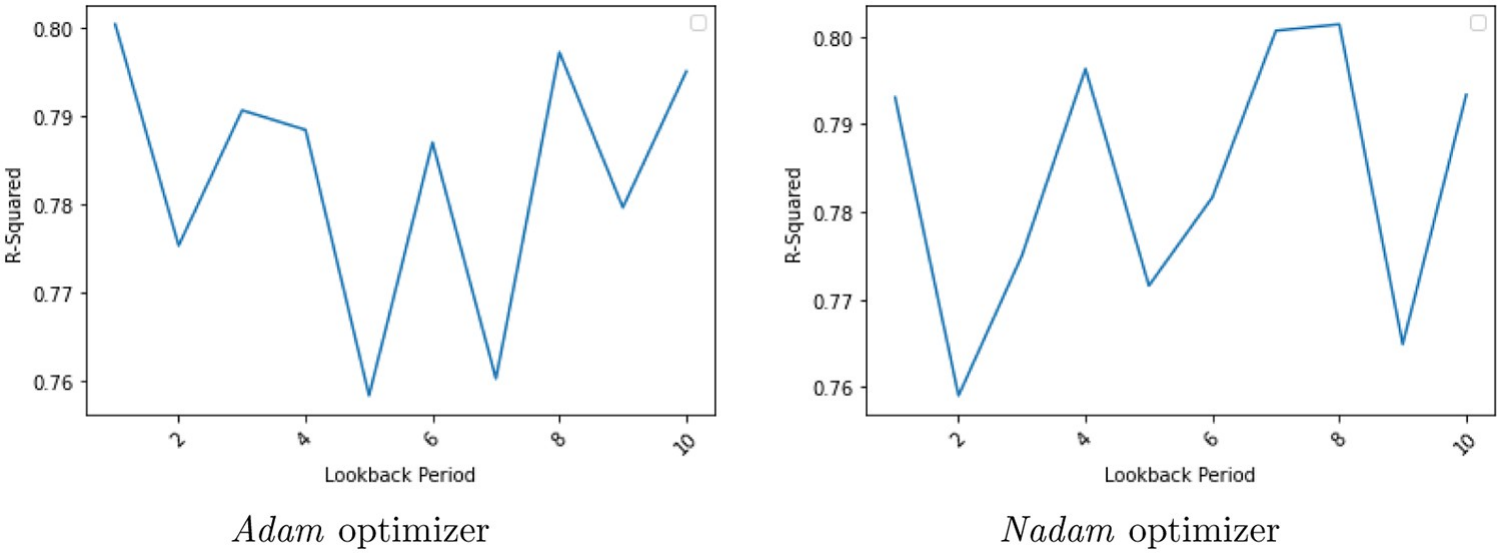
Lookback comparison of *Nadam* and *Adam* optimizers. We can see that the best R-squared is found corresponding to a lookback period of 1 day for the “*Adam*” optimizer and a lookback period of 8 days for the “*Nadam*” optimizer.

We use one LSTM layer, one dense layer, with the activation function of “tanh” and the loss was measured as “Mean Squared Error”. The training data and the test data are divided in 68:32 ratio. In Long Short-Term Method, the data have been scaled to a value between 0 and 1 using the min-max scalar value. We began with epoch = 70 and compared the lookback days from 0 to 10. This is done for both the “*Adam*” and “*Nadam*” optimizers. The results are shown in the Table 5. A question may arise as to why did we choose only *Adam* and *Nadam* to compare with. The answer lies in the fact that in this case, the predictive power when the LSTM is performed with other gradient descent optimizers is way below the *Adam* and *Nadam* optimizers. So we chose the *Adam* and *Nadam* optimizers. The graphs are shown in the Fig 14. We got a maximum R-squared corresponding to a lookback period of 1 day in the case of *Adam* and a lookback period of 8 days in the case of *Nadam*. In the case of *Adam*, it is 80.04% while in the case of *Nadam* it is 80.13%. Thereafter, we made a constant lookback period of 1 for *Adam* and 8 for *Nadam*, for our next optimization with respect to epochs.

**Table 5:** R-squared values corresponding to lookback periods for *Adam* and *Nadam* optimizers.

No. Of lag days	*Adam*	*Nadam*
1	0.8004	0.7930
2	0.7753	0.7590
3	0.7906	0.7750
4	0.7884	0.7963
5	0.7582	0.7715
6	0.7870	0.7816
7	0.7601	0.8006
8	0.7972	0.8013
9	0.7796	0.7648
10	0.7950	0.7933

**Fig 15:**
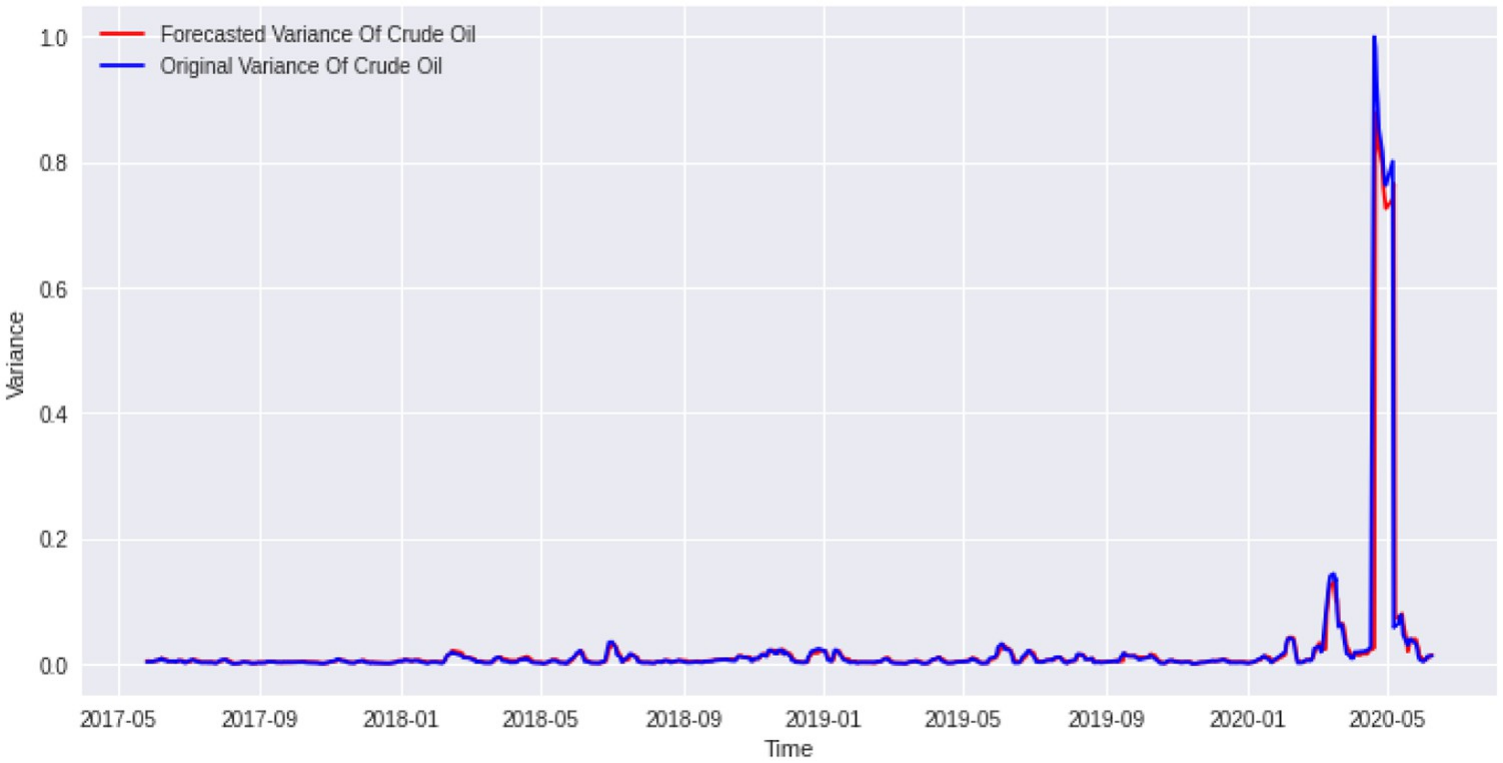
Forecasted values vs actual values of daily crude oil price variance from May 2017 to June 2020. The lookback period is 1 day. The number of epoch is 68. The activation function is ‘tanh’. and the gradient descent optimizer is “*Adam*”. It gives a R-squared of 80.18%. We can see that our model rightly predict the crude oil April 20, 2020, high volatility phase.

**Fig 16:**
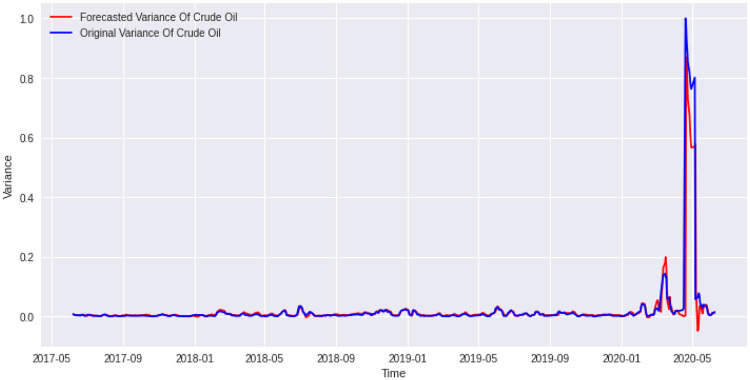
Forecasted values vs actual values of daily crude oil price variance from May 2017 to June 2020. The lookback period is 8 days. The number of epoch is 68. The activation function is ‘tanh’. and the gradient descent optimizer is “*Nadam*”. It gives a R-squared of 80.74%. We can see that our model rightly predict the crude oil April 20, 2020, high volatility phase.

Now, corresponding to a lookback period of 1 for *Adam* optimizer, we made a comparative analysis for epochs from 50 to 100. Similarly, corresponding to a lookback period of 8 for *Nadam* optimizer, we made a comparative analysis for epochs from 50 to 100. The results are shown in the Tables 6 and 7. The R squarred for the training data for *Adam* is 0.8944 while the same for *Nadam* is 0.8978. Coincidently, the best cases for both *Adam* and *Nadam* are found corresponding to an epoch of 68. We plotted a graph for the predicted value and the corresponding actual value for Crude Oil variances. The plots are given in Figs 15 and 16.

In the plots, we can see that the algorithm almost correctly predicted the actual values of variance. One of the most spectacular parts is that it could rightly predict price variance during the covid crisis. As a result, the *options traders* could use this algorithm to trade various crude oil options during these tumultuous times. Moreover, crude oil being important energy for human civilization, the governments could rightly make use of this algorithm to predict crude oil daily variance and make decisions. Besides that, we also found several new factors which influence crude oil daily variance and many other factors which do not influence the crude oil daily variance.

**Table 6:** *Adam* and *Nadam* epochs comparisons.

Epochs	*Adam 1* lookback period	*Nadam* 8 lookback Period
50	0.7973	0.7916
51	0.7907	0.7333
52	0.7715	0.7618
53	0.7905	0.7893
54	0.7738	0.7820
55	0.7988	0.7939
56	0.7971	0.7648
57	0.7920	0.7914
58	0.7897	0.7788
59	0.7821	0.7820
60	0.7914	0.7892
61	0.7954	0.7858
62	0.7937	0.7476
63	0.7937	0.7368
64	0.7958	0.8013
65	0.7841	0.7774
66	0.7786	0.7891
67	0.7806	0.7856
68	0.8018	0.8074
69	0.8008	0.7913
70	0.8004	0.8013
71	0.7941	0.7421
72	0.7951	0.7902
73	0.7874	0.7577
74	0.7966	0.7918
75	0.7983	0.7860
76	0.7730	0.7923
77	0.7973	0.7994
78	0.7970	0.7960
79	0.7921	0.7834
80	0.7982	0.7821
81	0.7935	0.7675
82	0.7967	0.7991
83	0.7919	0.7828
84	0.7896	0.7921
85	0.7994	0.7904
86	0.7715	0.7806

**Table 7:** *Adam* and *Nadam* epochs comparisons are continued from Table 8.

Epochs	*Adam* 1 day lookback period	*Nadam* 8 days lookback period
87	0.7931	0.7696
88	0.7994	0.7358
89	0.7878	0.7961
90	0.7599	0.7948
91	0.7938	0.7784
92	0.8033	0.7615
93	0.7669	0.7952
94	0.7971	0.7981
95	0.7929	0.7569
96	0.8001	0.7876
97	0.7828	0.7424
98	0.7824	0.7909
99	0.7861	0.7903
100	0.7910	0.7870

#### 5.3.1 Shapley values

We have calculated the **Shapley Values** of all the features for all the previous days, both for **Adam** and **Nadam**. The results are shown in Figs 17–25.

**Fig 17:**
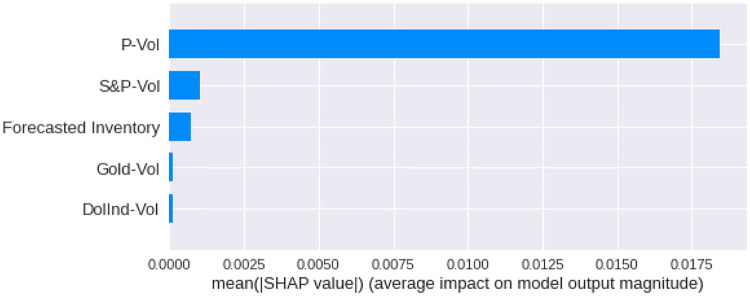
Mean Shapley values for the current day using Adam optimizer.

**Fig 18:**
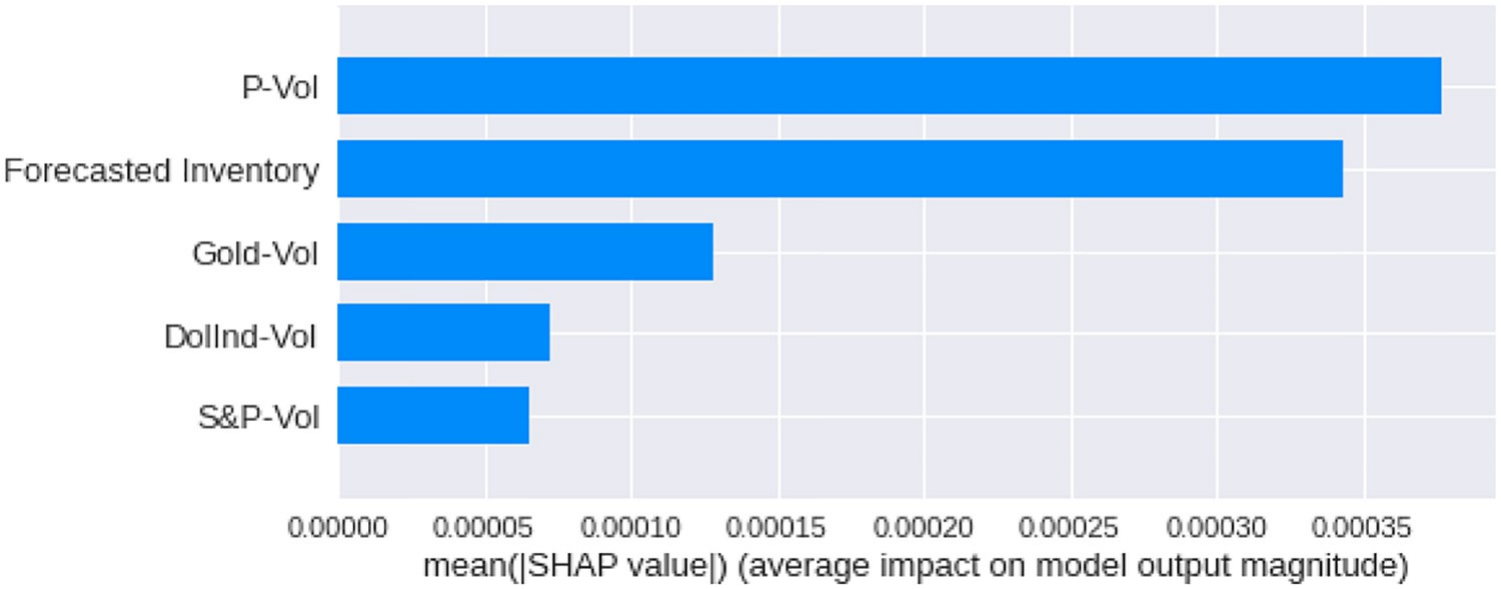
Mean Shapley values for the current day using Nadam optimizer.

**Fig 19:**
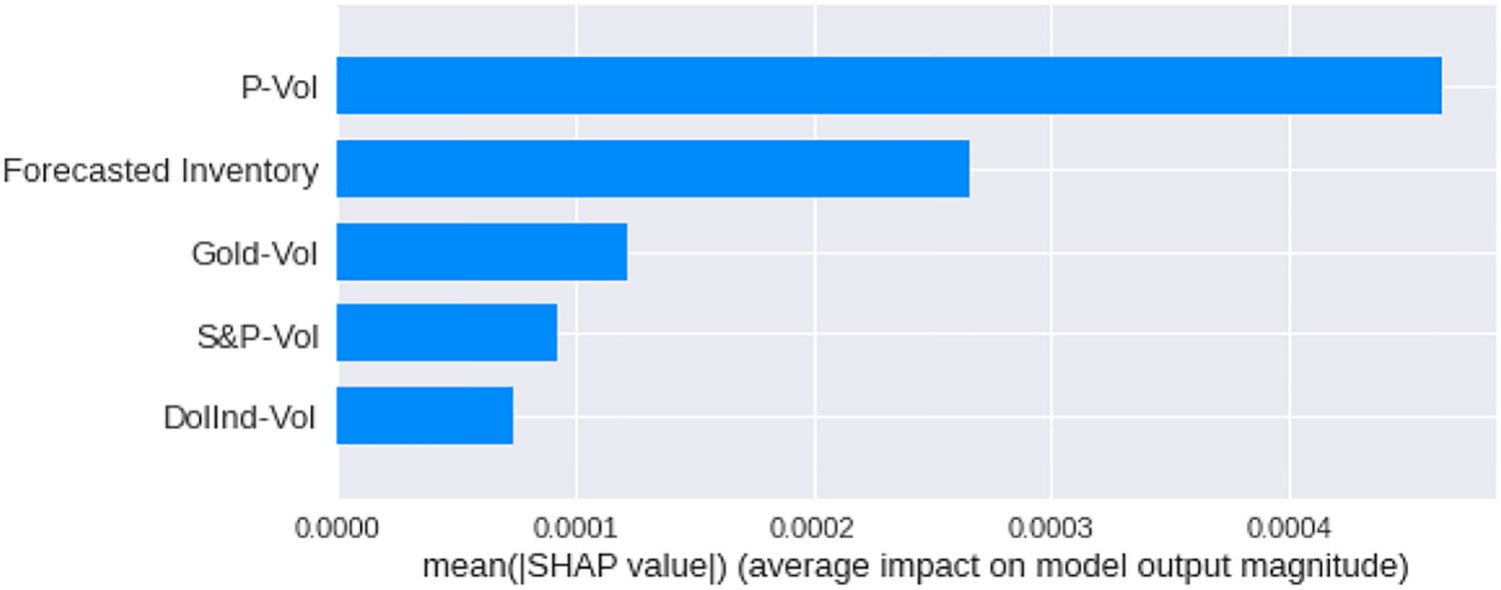
Mean Shapley values for the first previous day using Nadam optimizer.

**Fig 20:**
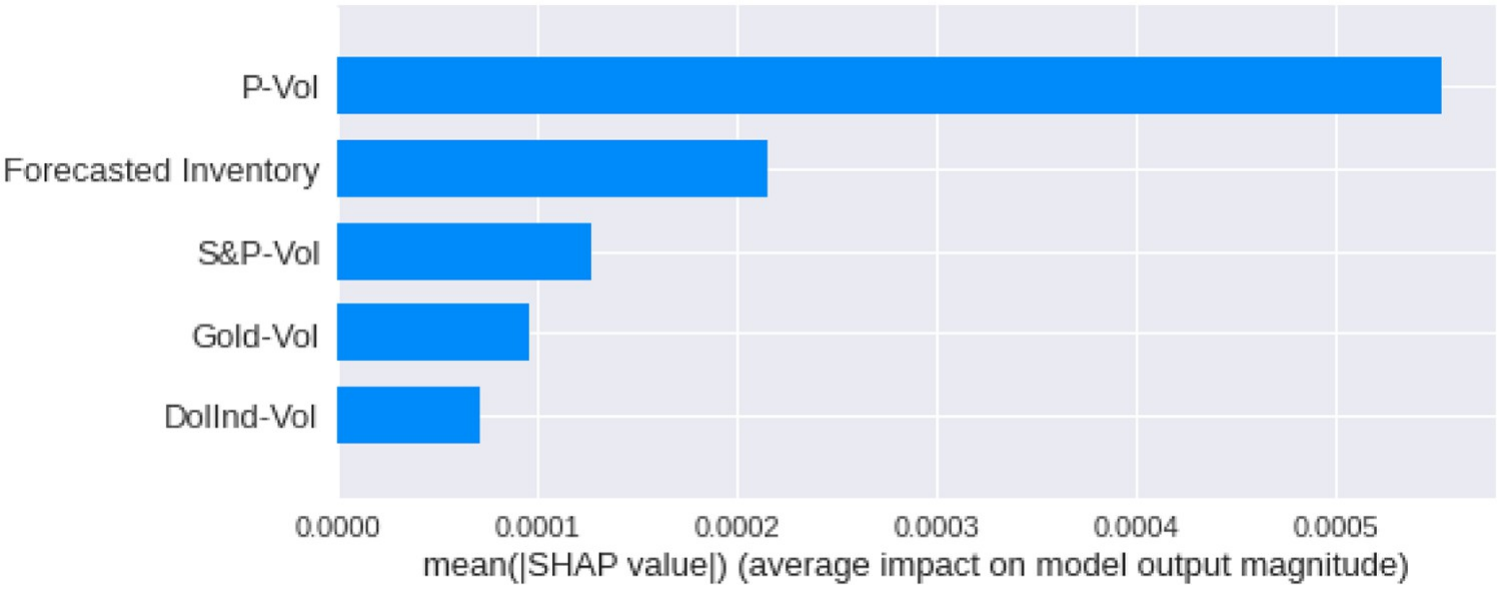
Mean Shapley values for the second previous day using Nadam optimizer.

**Fig 21:**
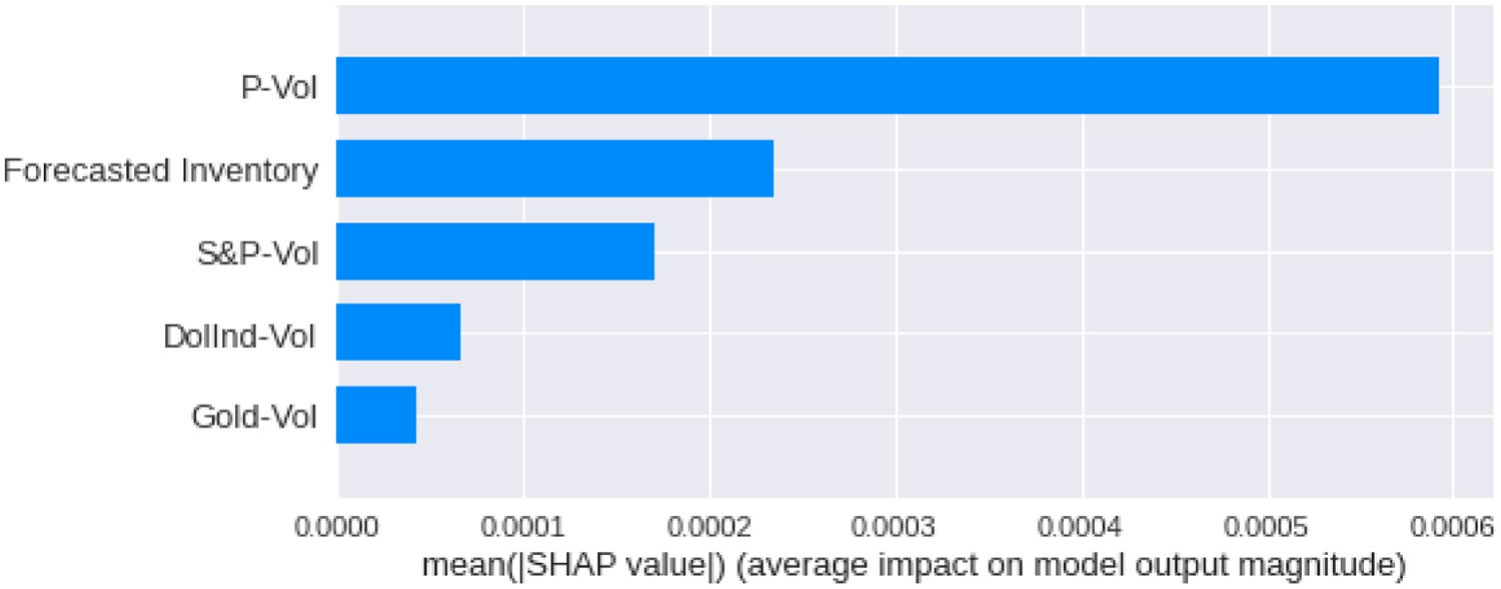
Mean Shapley values for the third previous day using Nadam optimizer.

**Fig 22:**
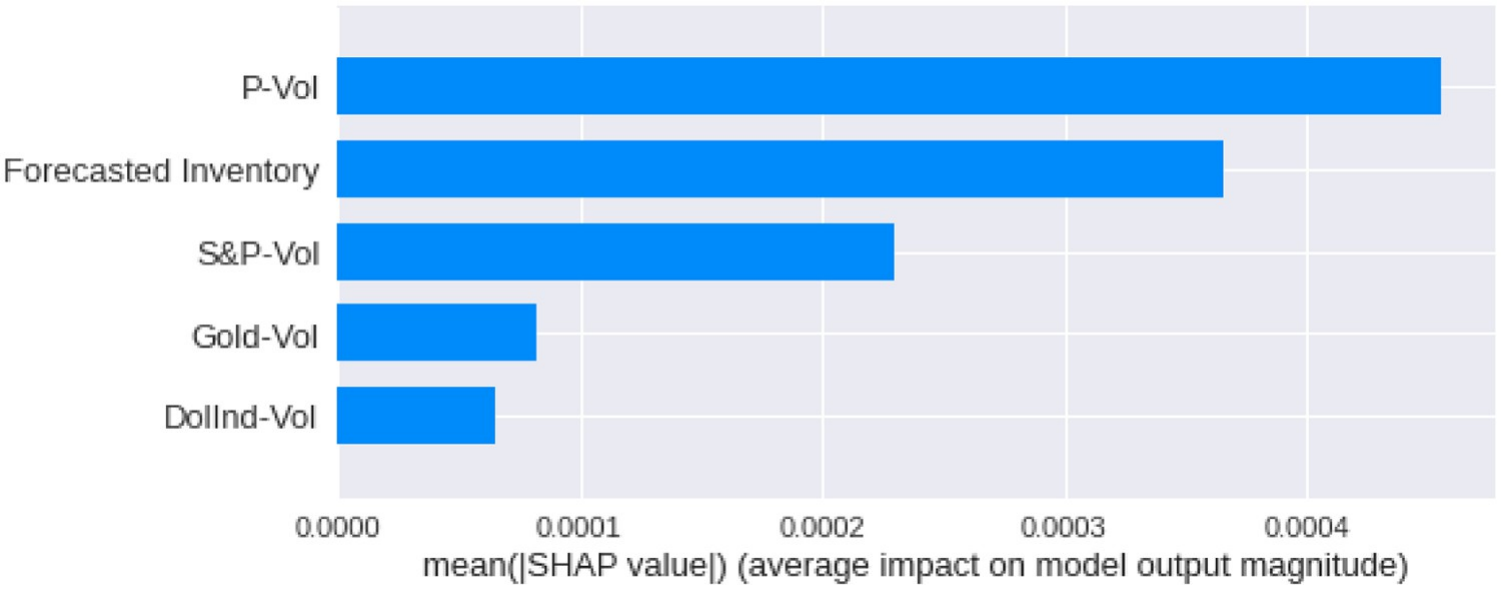
Mean Shapley values for the fourth previous day using Nadam optimizer.

**Fig 23:**
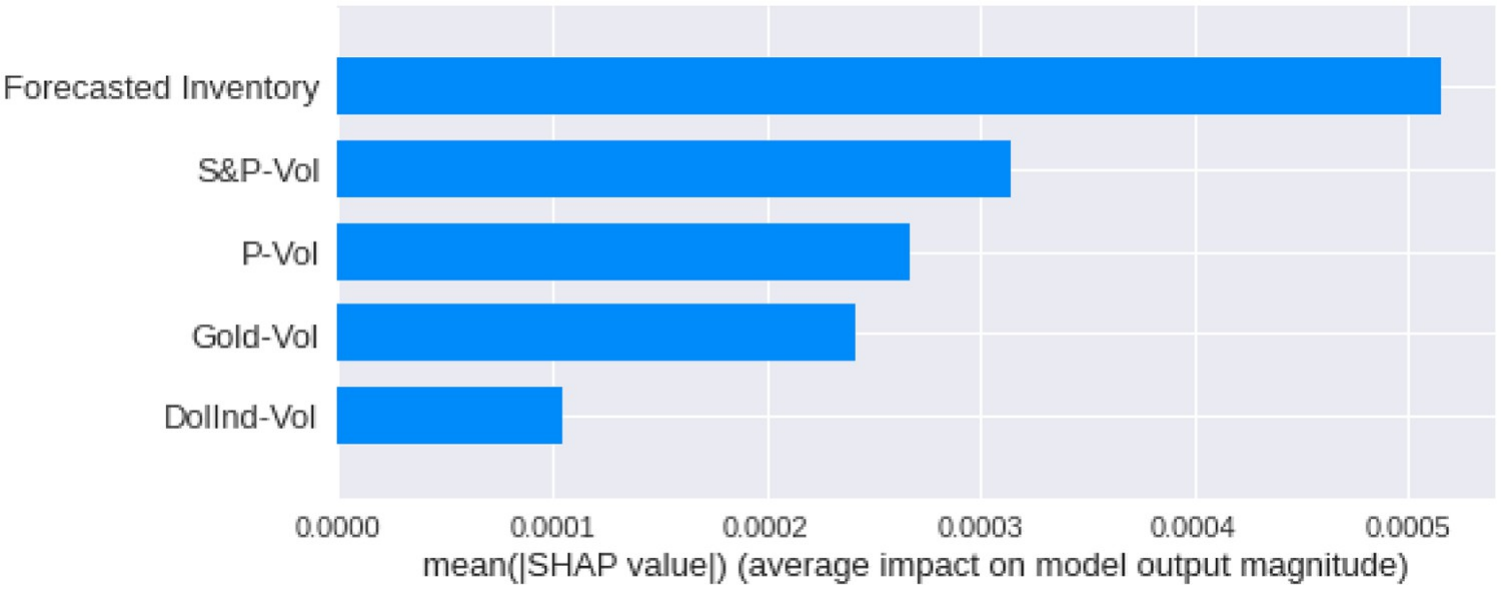
Mean Shapley values for the fifth previous day using Nadam optimizer.

**Fig 24:**
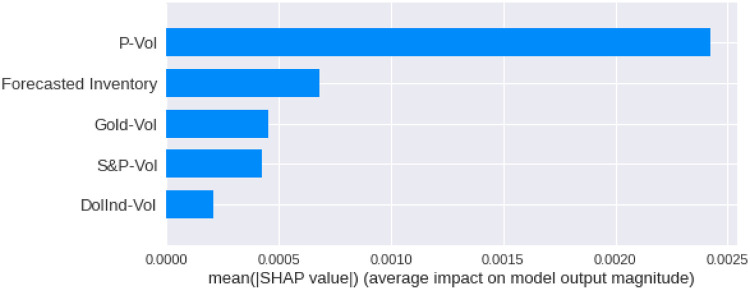
Mean Shapley values for the fifth previous day using Nadam optimizer.

**Fig 25:**
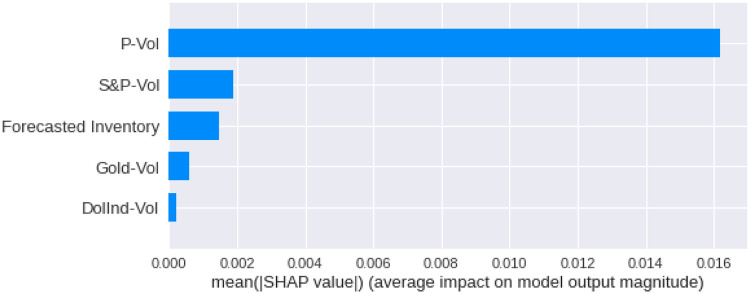
Mean Shapley values for the fifth previous day using Nadam optimizer.

A lot of research has been done to forecast the volatility of crude oil using several classical methods. We have made a comparative study regarding some of the latest research in this context. In some of them, Mean Squared Error (MSE) is given. For that, we have converted the MSE to Root Mean Square Error (RMSE). The values are given in Table 8 below. We can see that our model has the least RMSE.

**Table 8:** A comparison wrt. RMSE of four different models with our model.

Author	RMSE	Models Used
Ma, Liao, Zhang and Cao [71]	0.46	They used the mixed data sampling (MIDAS) modeling framework.
Zhang and Zhang [72]	0.17	They used a new hybrid forecasting method based on the hidden Markov, exponential generalized autoregressive conditional heteroskedasticity, and least squares support vector machine models is proposed
Abdollahi [16]	0.053	The author used a hybrid model consisting of complete ensemble empirical mode decomposition, support vector machine, particle swarm optimization, and Markov- switching generalized autoregressive conditional heteroskedasticity
Wei, Wang and Huang [73]	0.24	They used a linear and nonlinear generalized autoregressive conditional heteroskedasticity (GARCH) class models.
**Present Paper**	**0.04**	**We used a Multivariate-LSTM method.**

## 6 Conclusions

The variance of daily crude oil prices has significance on several factors. On a smaller scale, it is directly beneficial for the *options traders* who trade on the volatility of the underlying instrument. But on a larger scale, it is beneficial for the oil-importing countries, the oil-exporting countries, producers, oil miners, consumers, etc. Oil is one of the major energies for human civilization, and it is known to be highly fluctuating. So a thorough study of its prices is important.

The inventory level of any commodity is an important deciding factor for the price of that commodity. We studied the actual inventory, forecasted inventory, inventory shocks, etc., of crude oil, and studied how these factors could help us in forecasting the variance of daily oil price.

We then studied the influence of several factors on the crude oil variance. The volatility of Gold is famous for being related to Crude Oil volatility. Likewise, US bond yield and German Bund yield are also studied as they signify the risk-free interest. Bitcoin is one of the newest forms of digital currencies and it has several complications. It is banned in countries like China while it is accepted as a legal tender in countries like El Salvador. So we studied its price variance and its predictive power for crude oil variance. The US dollar plays a significant role as far as transactions in crude oil are concerned. For international transactions in Crude Oil, the US dollar is the medium of exchange. As such the variance of the Dollar index is also studied. S&P 500 is the stock index of the leading stock market in the USA, while Shanghai Stock Exchange is the leading stock market in China. As the USA and China are the two major economies, their stock indices hold an important position in determining global trade and commerce. So the variance of S&P 500 and SSE are also included.

With the help of Granger causality, we studied the effects of the variances of several factors on the variance of crude oil. We chose a few factors from among them which will help us to forecast the variance of crude oil prices. For forecasting, we used the LSTM method, which is one of the newly developed methods for time series analysis. After optimizing the hyperparameters we got an R-squared value of 80.74%.

We can derive several important conclusions from our present work. To begin with, compared to 2006-2011, in the last decade 2011-2020, the forecasted inventory still has substantial predictive power over actual inventory. The forecasted inventory value has a huge impact on the variance of the daily crude oil prices, while the absolute value of the inventory information shock has no influence over the daily crude oil variance. We found out that past values of crude oil, forecasted inventory, variances of S&P 500, Dollar index, and Gold Granger cause crude oil. Thereafter, we found out that these values can explain the variation of the variances of daily crude oil prices to the extent of 80.74%. We could see that our method could successfully predict the variance in the oil crash during COVID, for the month of April 2020.

The results derived based on our present work can be presented pointwise below:

- Forecasted inventory can still regress actual inventory with an adjusted R-squared value of 0.343 for the time period of 2011-2020. Hui found that forecasted inventory regressed actual inventory for the time period of 2006-2011 with an R-squared value of 0.1926. The DW test result found by Hui is 1.9589 and while that of ours is 2.085. The correlation coefficient between forecasted inventory and actual inventory found by Hui is 0.4412 whereas the value found by us is 0.5871.
- The difference between Hui and our research is that Hui studied the conditional volatility of daily returns, whereas we studied the variance of daily Crude Oil prices. Another difference is that Hui primarily focussed on statistical methods to do the analysis, while we focussed on deep learning methods like LSTM for the same analysis.
- We found out that daily variance of Bitcoin, Shanghai Stock Exchange, German 10 Year Bund yield, and US 10 Year bond yield do not granger cause variance of daily crude oil prices.
- Likewise the daily variance of Bitcoin, Shanghai Stock Exchange, German 10 Year Bund yield, and US 10 Year bond yield do not granger cause variance of daily crude oil prices.
- The daily variance of Bitcoin, Shanghai Stock Exchange, German 10 Year Bund yield, and US 10 Year bond yield do not granger cause variance of daily crude oil prices.
- The forecasted inventory, and variance of S&P 500 granger cause variance of daily crude oil prices. Variances of gold and Dollar index granger cause variance of crude oil after several days.
- We also found that an LSTM strategy of one LSTM layer and one dense layer can forecast the variance of crude oil when forecasted inventory, variances of previous day crude oil, S&P 500, the Dollar index, and gold are taken as independent variables.
- After optimizing the various hyperparameters, we got that the maximum accuracy is found for the *Adam* optimizer when we take a lookback of 1 day, epochs = 68, activation function = tanh. The R- squared value is equal to 80.18%. This strategy could predict the high variance of oil prices during the COVID crisis of crude oil.
- After optimizing the various hyperparameters, we got that the maximum accuracy is found for the *Nadam* optimizer when we take a lookback of 8 days, epochs = 68, activation function = tanh. The R- squared value is equal to 80.74%. This strategy could predict the high variance of oil prices during the COVID crisis.

## Supporting information

S1 Data (CSV)
